# Mapping diversity in African trypanosomes using high resolution spatial proteomics

**DOI:** 10.1038/s41467-023-40125-z

**Published:** 2023-07-21

**Authors:** Nicola M. Moloney, Konstantin Barylyuk, Eelco Tromer, Oliver M. Crook, Lisa M. Breckels, Kathryn S. Lilley, Ross F. Waller, Paula MacGregor

**Affiliations:** 1 Department of Biochemistry, University of Cambridge, Cambridge, CB2 1QW UK; 2 Cell Biochemistry, Groningen Biomolecular Sciences and Biotechnology Institute, University of Groningen, 9747 AG Groningen, Netherlands; 3 Department of Statistics, University of Oxford, Oxford, OX1 3LB UK; 4 School of Biological Sciences, University of Bristol, Bristol, BS8 1TQ UK

**Keywords:** Organelles, Proteomics, Parasitology

## Abstract

African trypanosomes are dixenous eukaryotic parasites that impose a significant human and veterinary disease burden on sub-Saharan Africa. Diversity between species and life-cycle stages is concomitant with distinct host and tissue tropisms within this group. Here, the spatial proteomes of two African trypanosome species, *Trypanosoma brucei* and *Trypanosoma congolense*, are mapped across two life-stages. The four resulting datasets provide evidence of expression of approximately 5500 proteins per cell-type. Over 2500 proteins per cell-type are classified to specific subcellular compartments, providing four comprehensive spatial proteomes. Comparative analysis reveals key routes of parasitic adaptation to different biological niches and provides insight into the molecular basis for diversity within and between these pathogen species.

## Abstract (web summary)

The molecular diversity between parasite species and life-stages correlate with diversity in the hosts they infect or the pathologies they cause. Here, Moloney et al., map the spatial proteomes of two African trypanosome species across two life stages and identify key routes of parasitic adaptation.

## Introduction

Kinetoplastids are single-celled, flagellated eukaryotes that include important parasites of humans, livestock and crop plant species and are typically transmitted by invertebrates. Within this class are the African trypanosomes, which collectively infect a range of mammals and cause Human and Animal African trypanosomiasis. The majority of research characterising African trypanosomes has been performed with *Trypanosoma brucei*; partly because two sub-species are human infective, also, the relative ease of in vitro culture and genetic manipulation of this species. *T. brucei* has been studied as a parasite, but also as a divergent model organism, with both well-conserved and non-canonical eukaryotic biological features of interest. The related species, *Trypanosoma congolense* and *Trypanosoma vivax* are the main causative agents of cattle trypanosomiasis. Despite their veterinary importance, considerably less research has occurred on these species^1^. Different African trypanosome species have distinct cellular and infection characteristics, yet the molecular basis of much of this is unknown^2–5^.

African trypanosomes are exposed to a range of different external environments during their life cycle and the parasites differentiate between a series of life-stages that are each adapted for growth and survival in their current environment or pre-adapted for the next^6^. Each life-stage is based on a common, highly organised, polar core cellular architecture, with a single flagellum and a collection of single- and multi-copy organelles. The relative sizes, positions and protein contents of organelles vary between life-stages. As in all eukaryotic cells, the subcellular localisation of a protein in trypanosomes not only defines the biochemical environment of that protein, but also the potential for molecular interactions. Hence, protein function is intimately linked with protein localisation.

There are two main approaches in determining protein localisation in a cell: microscopy and proteomics. Microscopy allows for precise resolution of specific localisations; can detect variation between cells within a sample; and can readily identify proteins localised to multiple sites, although can suffer from artefacts of tagging or inappropriate expression. Due to the requirement to obtain a protein-specific antibody or to genetically manipulate the protein of interest, microscopy is generally limited to small numbers of proteins per study. Proteome-wide microscope analyses are valuable, rich datasets but non-trivial and time-consuming endeavours which are so far limited to few species: *Saccharomyces cerevisiae*^7^, Humans^8^ and *T. brucei*^9^.

Spatial proteomics, based on the isolation or enrichment of organelles followed by mass spectrometry (MS), allows for the identification of proteins enriched in specific subcellular locations - usually without the need for genetic modification. These methods have been highly effective in revealing protein residents of organelles or structures within trypanosomatids, such as the mitochondrion, glycosomes, flagellum and nucleus^10–14^. High-throughput MS-based methods can now be used to systematically localise thousands of proteins in a single experiment for multiple conditions, states, or cell types^15–22^. Such methods include hyperLOPIT (hyperplexed localisation of organelle proteins by isotope tagging)^16,23^. This is a quantitative proteomics approach whereby a spatial map of the proteome can be resolved without the need for isolation of individual organelles enabled by the application of machine learning algorithms^24–27^. HyperLOPIT, and related LOPIT methodologies, have been utilised to generate spatial maps of mammalian, insect, yeast, plant and protozoan cell-types^16,21,28–32^.

Here, hyperLOPIT, combined with computational modelling, has been employed to spatially map the proteomes of *T. brucei* and *T. congolense*, each in two different life-stages: the mammalian bloodstream form (BSF) and the insect midgut procyclic form (PCF)^23,33^. This has resulted in four comprehensive and complementary datasets that (i) individually provide evidence of protein expression and assignment of subcellular location of thousands of proteins per cell-type, and (ii) collectively facilitate comparative analysis between life-stages and species. This has highlighted key subcellular locations in parasitic adaptation to biological niches and the molecular basis for the diversity observed within and between these distinct pathogen species.

## Results

### Implementation of the hyperLOPIT method in African trypanosomes yields global coverage and resolution of the proteome

**Fig. 1:**
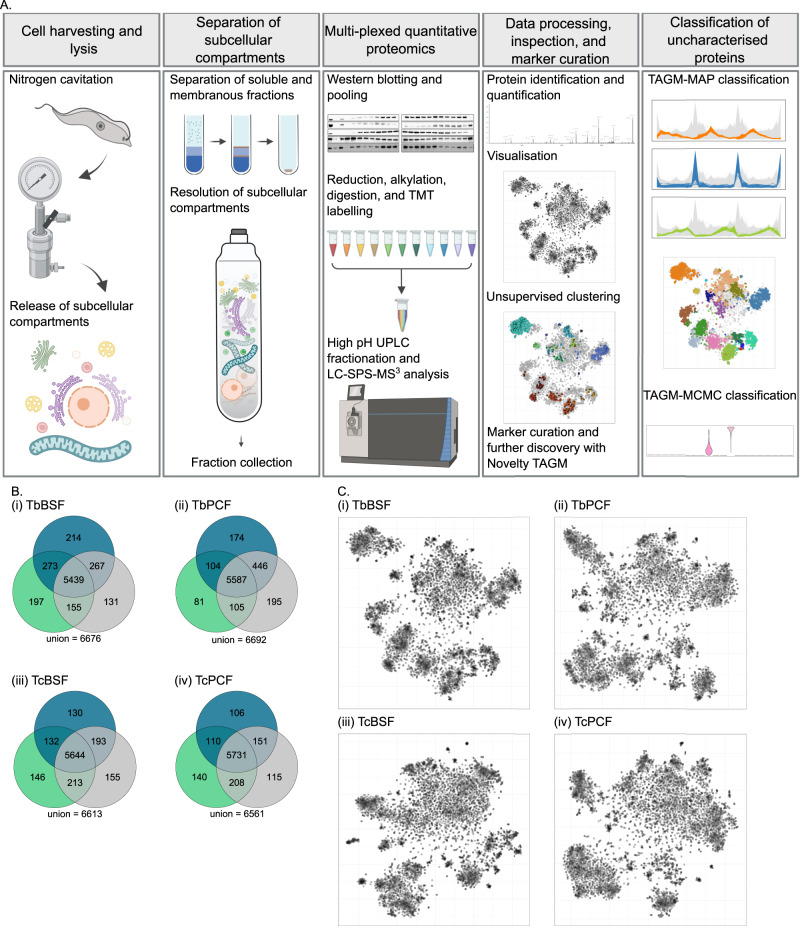
HyperLOPIT was employed to resolve the spatial proteome of *T*. *brucei*and *T. congolense* BSF and PCF. **A** Outline of the experimental workflow to implement hyperLOPIT on African trypanosomes: first cells were harvested and lysed by nitrogen cavitation releasing subcellular compartments. Soluble proteins and crude membranes were separated on an iodixanol cushion followed by pelleting of the membranous fraction, which was then resolved by density gradient centrifugation. After collection, fractions were analysed by western blotting, strategically pooled and prepared for multiplexed quantitative proteomics with LC-SPS-MS^3^ analysis. Peptide identification and quantification were performed in ProteomeDiscoverer with further processing including aggregation and normalisation performed in R. Datasets were inspected visually (t-SNE) and with unsupervised clustering (HDBSCAN). Unsupervised clustering guided the curation of marker proteins allied with Novelty-TAGM for further discovery in *T. congolense*. Resultant marker proteins were used in the classification of uncharacterised proteins using TAGM-MAP. Further analysis was performed with TAGM-MCMC for insight into proteins unclassified with TAGM-MAP that may reside in multiple locations. Image created with BioRender.com. **B** Three hyperLOPIT experimental iterations (#1–3) were conducted and data from all iterations were concatenated per cell type. Venn diagrams show that the final datasets contain approximately 5500 proteins per cell-type for which there is 33-plex quantitative data. **C** Dimensionality reduction via t-SNE projection facilitates visualisation of each 33-dimensional dataset in two-dimensions which reveals structure within the data. Each point represents a protein.

The experimental workflow for hyperLOPIT in African trypanosomes (Fig. 1A) was developed based on previous work^23,28^. Cells were lysed by nitrogen cavitation^10,34^. Crude membranes and soluble proteins were then separated on an iodixanol cushion with membranes subsequently collected and pelleted by ultracentrifugation. Membranes were resolved by density gradient centrifugation on an iodixanol gradient. Approximately 22 fractions were collected from the gradient and first assessed by western blot to confirm compartment resolution (Supplementary Fig. 1). Fractions were pooled to 10 per experiment, which, in addition to the cytosolic-enriched soluble fraction, formed the basis for 11-plex TMT (tandem mass tag) labelling. TMT-labelled fractions were combined, fractionated by high pH UPLC and subjected to multiplexed quantitative MS with LC-SPS-MS^3^.

In spatial proteomics, often no individual experiment achieves optimal subcellular compartment resolution, however, multiple experiments in combination can collectively improve compartment resolution and protein localisation^35^. Hence, for each of the four cell-types, three experimental iterations were carried out. In each case, the cell lysis and fraction pooling methods were varied between each of the three independent experiments (Supplementary Data 1), with a view to improve overall resolution once the data were combined. 6561-6692 proteins were identified across the three experimental iterations and quantifications from each of the 11-plex experiments were concatenated (creating a 33-plex dataset; see Supplementary Note). Principal component analysis was used to demonstrate that, despite deliberate variation in experimental method between the experiments, the 11-plex fractions tend to cluster together across experiments indicating good reproducibility of the fractionation method (Supplementary Fig. 2)^36,37^. Proteins missing in any iteration per cell-type were removed. This resulted in 5439–5731 proteins for spatial proteome characterisation (Fig. 1B and Supplementary Data 2). After normalisation, each 33-dimensional dataset was projected in two dimensions by t-SNE (t-distributed stochastic neighbour embedding) for visualisation (Fig. 1C)^38^. This revealed structure within each dataset with resolution of discrete collections of proteins according to similarity in abundance profiles on the density gradient which, in turn, would be expected to have similar subcellular localisations^39^.

### Resolution within the spatial proteomes represents subcellular locations

Next, the data structure was examined to determine if it corresponded to resolution of subcellular niches. First, the abundance distribution profiles of selected functionally related groups of proteins or protein complexes were visualised in *T. brucei* BSF and PCF (Supplementary Fig. 3 and Supplementary Data 3). Protein functional cohorts showed clear evidence of common distinct distributions across the gradient. These distributions indicate differential resolution of functionally related proteins within the *T. brucei* datasets consistent with their spatial organisation.

**Fig. 2:**
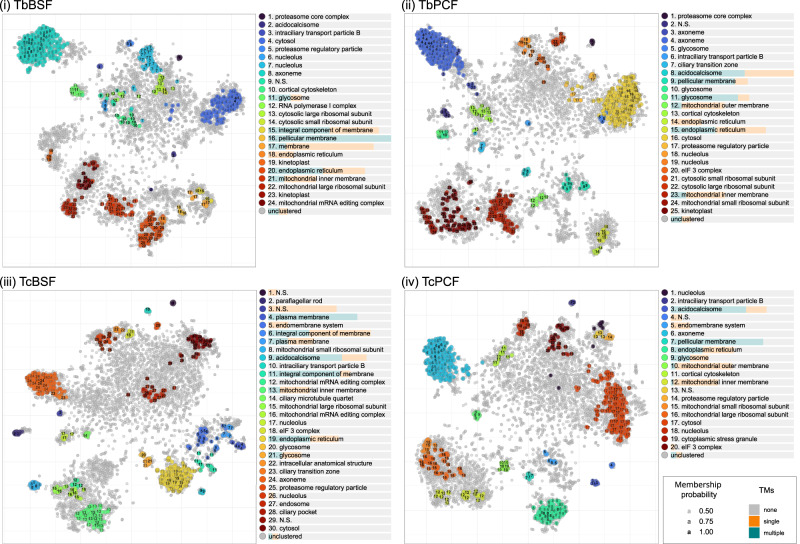
Feature analysis of unsupervised clusters generated with HDBSCAN demonstrates resolution of distinct subcellular functional compartments in each dataset. HDBSCAN clusters were highlighted on t-SNE projections for (i) *T. brucei* BSF, (ii) *T. brucei* PCF, (iii) *T. congolense* BSF and (iv) *T. congolense* PCF. Significantly represented GO CC terms in each cluster are displayed in the legend associated with each t-SNE projection (N.S. = not significant, with no significantly represented terms). The proportional representation of predicted single or multiple TM domains among proteins in each cluster is displayed below each cluster name in the associated legend.

To assess the spatial resolution more globally, and in a data-driven manner, the datasets were subjected to unsupervised clustering using HDBSCAN (Hierarchical Density-Based Spatial Clustering of Applications with Noise)^28,40^. In this algorithm, the similarity of protein abundance profiles across the 33 TMT channels (corresponding to three experiments) were evaluated. Each resulting cluster represents a set of proteins collectively well resolved along the density gradient, which in turn, likely represent discrete subcellular niches. HDBSCAN analysis revealed 20-30 clusters collectively comprised of 1144-1302 proteins per cell-type with 9–417 proteins per cluster as highlighted on t-SNE projections (Fig. 2). GO CC (Gene Ontology Cellular Component) enrichment analysis confirmed the clusters represent a diverse set of relevant biological niches within the cell (Fig. 2). In some cases, sub-compartment resolution was also observed, for example there was resolution of membranous and soluble mitochondrial proteins in each cell-type (Fig. 2). The contents of each HDBSCAN cluster were also analysed for protein features, including pI (isoelectric point), TM (transmembrane) domains, signal peptides and GPI (glycosylphosphatidylinositol) anchors (Supplementary Fig. 4 and Supplementary Data 4)^41–44^. The biophysical properties of the clusters exhibited clear and rational differential resolution. For example, clusters enriched with mitochondrial proteins showed an alkaline shifted pI, whereas clusters enriched with cytosol proteins showed an acidic shifted pI (Supplementary Fig. 4A)^45^. Proteins with predicted TM domains were in clusters associated with related GO CC terms, such as the mitochondrial membranes, ER (endoplasmic reticulum) and pellicular membrane (Fig. 2). Proteins predicted to contain signal peptides or GPI-anchors were primarily in clusters associated with GO CC terms such as ‘endomembrane system’ (Supplementary Fig. 4B). Collectively these analyses demonstrate resolution of diverse subcellular compartments with relevant composite biochemical protein features in accordance with expectations within each dataset.

### Machine-learning methods classify thousands of proteins per cell type to subcellular locations

The spatial proteomes were next characterised using semi-supervised machine learning approaches. Various methods have been developed for use with LOPIT data to classify unlabelled proteins to a set of compartments to define the spatial proteome^25–27^. Here this was performed using the TAGM (t-augmented Gaussian mixture) model with the TAGM-MAP (*maximum a posteriori*) method. This computes the probability of a protein belonging to one of the pre-defined subcellular compartments and an outlier component, which can be used for the basis of protein classification. To conduct this analysis, marker proteins collectively representing the compartments of the cell were first required.

*T. brucei* markers were selected based on a literature review and GO CC annotation, and guided by the HDBSCAN clustering, with exclusion of outlier proteins or those differentially localised between BSF and PCF (Supplementary Fig. 5A and Supplementary Data 5). *T. congolense* is less well studied so this approach was not feasible. Instead, an initial marker set was developed based on orthologs of *T. brucei* markers or TAGM-MAP allocations and guided by HDBSCAN clustering. As differences between *T. brucei* and *T. congolense* could result in unrepresented but resolved biological niches, the semi-supervised Bayesian method, Novelty TAGM, was also performed using this initial marker set (Supplementary Data 5-6)^46^. This algorithm enables discovery of unlabelled compartments in addition to allocating proteins to defined compartments. The resulting allocations were used to define separately resolved compartments in *T. congolense* and guide expansion of markers in existing compartments (Supplementary Fig. 6A). A total of 891 *T. brucei* marker proteins were selected, with 852 and 849 proteins representing 20 distinct subcellular niches in the BSF and PCF, respectively (Supplementary Fig. 5B, C, Table 1 and Supplementary Data 5 and 7). For *T. congolense*, a total of 734 marker proteins were selected, with 719 and 713 proteins representing 23 and 19 distinct subcellular niches in BSFs and PCFs, respectively (Supplementary Fig. 6B, C, Table 1 and Supplementary Data 5 and 7).

**Table 1:** Summary of subcellular compartments represented in the spatial proteomes for each cell-type. An extended version of Table 1 is available in Supplementary Data 7

Compartment	Description	Examples^a^	Compartment grouping^b^
Acidocalcisomes	Small membranous acidified vacuoles present in the cytoplasm.	VIT1, VTC1, VTC4	Acidocalcisomes
Cytosol	The intracellular contents of the cell within the bounds of the plasma membrane not otherwise contained in organelles or the cytoskeleton.	ECT, GMPS, NBP2, pdxK	Cytosol
Endoplasmic reticulum (ER)	The membrane and lumen of the membranous network of the ER including sub-domains in addition to nuclear envelope. Compartment set includes ER and nuclear envelope proteins.	DPMS, ELO1, GPI16, SPT	Endoplasmic reticulum (ER)
Flagellum 1	Core cytoskeletal components of the flagellum including the axoneme and paraflagellar rod.	CARP1, CMF10, DRC4, PFC12, SAXO1, TRYPARP	Flagellum
Flagellum 2	A subset of the flagellum 1 compartment set separately resolved in TcBSF only.	PFC1, PFC7	Flagellum
Glycosomes	Small membranous peroxisome-like organelles containing glycosomal matrix and membrane proteins.	GAT1, PEX10, TFEalpha1	Glycosomes
Intraflagellar transport	Intraflagellar transport proteins which comprise a set of proteins involved in the construction of the flagellum.	IFT46, IFT52, PIFTB2	Intraflagellar transport
Microtubule structures 1	Cytoskeletal proteins associated with flagellar and microtubule proximal structures.	BOH1, POC11	Microtubule structures 1
Microtubule structures 2	Cytoskeleton interacting proteins including those involved in cytoskeleton organisation, microtubule-based movement and cell division.	KAT60A, KIN13-5, KH1	Microtubule structures 2
Mitochondrion - matrix 1	Mitochondrial matrix including ribosomal proteins.	MRPS6, MRPS11, KRIPP8	Mitochondrion - matrix
Mitochondrion - matrix 2	Mitochondrial matrix including proteins involved in matrix-localised processes, i.e. RNA editing and iron-sulfur cluster assembly and ribosomal proteins in TcPCF.	FHm, KREPA1, PAMC1, NFU1, TOP2	Mitochondrion - matrix
Mitochondrion - matrix 3	Mitochondrial matrix including large ribosomal proteins separately resolved in TcBSF only.	MRPL2, MRPL15	Mitochondrion - matrix
Mitochondrion – inner membrane (IM)	Mitochondrial IM including components of the electron transport chain and mitochondrial inner membrane import machinery.	AOX, TIM62, SDH5, MIC10-1	Mitochondrion - membranes
Mitochondrion – outer membrane (OM)	Mitochondrial OM including components of the mitochondrial outer membrane import machinery.	ATOM40, POMP1	Mitochondrion - membranes
Nucleus	Nucleolar proteins as well as non-chromatin components of the nucleus.	NOP105, U2AF35, KKIP2	Nucleus
Nucleus – chromatin	Chromatin component of nucleus including DNA interacting proteins.	CITFA-4, H3V, ISWI, NLP	Nucleus – chromatin
Proteasome	Alpha and beta sub-units of the proteasome.	PSA4, PSB4	Proteasome
Proteasome regulatory particle	Regulatory particle sub-units of the proteasome.	RPN1, RPT2	Proteasome regulatory particle
Ribonucleoproteins 1	Ribosomal proteins and translation initiation factors. In TbBSF, this also included stress granule proteins.	40S and 60S ribosomal proteins	Ribonucleoproteins
Ribonucleoproteins 2	A subset of 60 S ribosomal proteins was separately resolved in TcBSF only.	60S ribosomal protein	Ribonucleoproteins
Secretory/ endocytic 1	Secretory pathway, surface and endocytic compartments, including trafficking, golgi, putative surface, endosomal, and lysosomal proteins.	ABCG5, GLP1, syntaxin 5, VCL2, VCL3	Secretory/ endocytic
Secretory/ endocytic 2	Secretory pathway, surface and endosomal compartments, including putative surface transporters.	AQPs, AA transporters, glucose transporters	Secretory/ endocytic
Secretory/ endocytic 3	Secretory pathway, surface and endosomal compartments, including putative surface and endosomal proteins.	ISG64 (TbBSF), ACP3 (TbPCF), VSG domain proteins (TcBSF)	Secretory/ endocytic

^a^ Protein names based on TriTrypDB annotation of *T. brucei* genes.

^b^ Grouped compartment name for downstream comparative analysis.

**Fig. 3:**
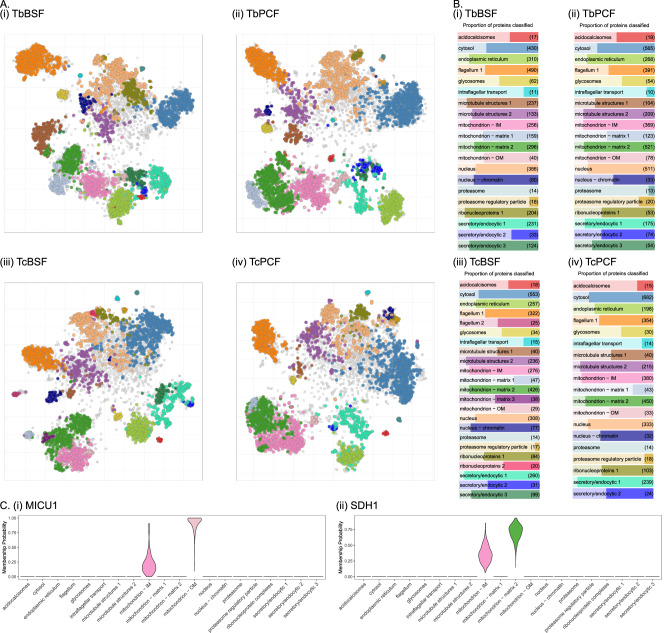
TAGM classification of uncharacterised proteins to subcellular compartments based on curated marker proteins. **A** Marker proteins and TAGM-MAP classifications highlighted on t-SNE projections for (i) *T. brucei* BSF, (ii) *T. brucei* PCF, (iii) *T. congolense* BSF and (iv) *T. congolense* PCF (compartment colours are as indicated in panel **B**). **B** Marker proteins (transparent area) and TAGM-MAP classifications of unlabelled proteins (opaque area) visualised on bar-plots proportionally, grouped according to compartment for indicated cell-type. Total number of proteins is indicated on the right. **C** Violin plots showing distribution of localisation probabilities across each subcellular compartment according to TAGM-MCMC analysis in *T. brucei* PCF for (i) MICU1 and (ii) SDH1.

Next, the classification of unlabelled proteins was performed using TAGM-MAP to assign proteins to subcellular compartments (Fig. 1A and Supplementary Data 8 and 9). Each protein was probabilistically assigned a compartment allocation. Allocations represent the most probable compartment localisation of every protein within the dataset. Protein classifications are generated by applying a threshold to these allocations, with proteins that do not meet the criteria designated as ‘unknown’. To the assess reproducibility of outcome classifications between individual experimental iterations within cell-types, classifications were performed on each individual 11-plex dataset (prior to concatenation into their respective 33-plex datasets). Individual datasets were compared pairwise using the adjusted Rand index and indicated good consistency ( > 0.85 for all pairwise comparisons), demonstrating the reproducibility between experimental iterations (Supplementary Fig. 7 and Supplementary Data 8)^47^. Next, to define the spatial proteomes for each cell-type, final classifications were generated by performing TAGM-MAP analysis on each combined 33-plex dataset (Fig. 1A and Supplementary Data 9). Using this approach, 2679 and 2795 proteins were classified in *T. brucei* BSF and PCF respectively (Fig. 3A, B and Supplementary Data 9). In *T. congolense*, 2507 and 2504 proteins were classified in the BSF and PCF respectively (Fig. 3A, B and Supplementary Data 9). These four spatial proteomes provide a comprehensive localisation dataset for two closely related species, across two life-stages each, which has not been achieved on this scale before in any parasitic organism.

**Fig. 4:**
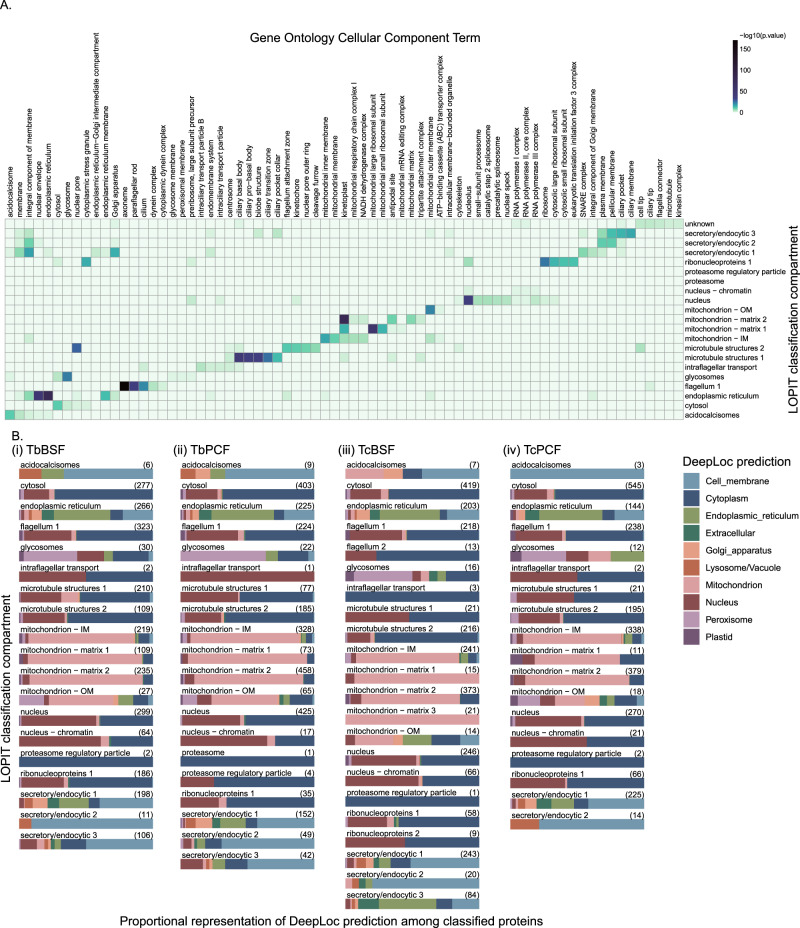
Correlation of TAGM-MAP classifications with orthogonal approaches. **A** Heatmap of GO CC term representation in TAGM-MAP classifications for *T. brucei* BSF. hyperLOPIT compartments are on the Y-axis, GO CC terms are on the X-axis, colours are scaled by the −log10 (*p*-value) for the over-representation of GO CC terms in the indicated compartment versus the background proteome. **B** DeepLoc subcellular compartment predictions visualised with bar-plots proportionally, grouped according to compartment for (i) *T. brucei* BSF, (ii) *T. brucei* PCF, (iii) *T. congolense* BSF and (iv) *T. congolense* PCF. Total number of classified proteins for each compartment is indicated on the right.

To interrogate the fidelity of and validate the TAGM-MAP classifications, the four datasets were inspected using alternative localisation annotations. For each subcellular location, classified proteins were subjected to GO CC term enrichment analysis (Fig. 4A and Supplementary Fig. 8). The GO CC term annotation includes curated localisations based on experimental studies^43^. This revealed clear and sensible patterns for all compartments in all cell-types. For example, flagellum 1 was enriched with terms including ‘axoneme’ and ‘paraflagellar rod’, while secretory/endocytic 1-3 were enriched with terms including ‘pellicular membrane’, ‘Golgi apparatus’ and ‘ciliary pocket’. The subcellular localisation of proteins was also compared with sequence-based localisation predictions using DeepLoc (Fig. 4B and Supplementary Data 4)^48^. Predictions for proteins classified to each subcellular compartment in the four datasets showed clear agreement in protein localisations. As examples: 66–73% of cytosol classifications showed a DeepLoc prediction of cytoplasm, and 74–81% of the collective mitochondrial classifications (mitochondrion – OM (outer membrane), IM (inner membrane), and matrix 1, 2 and 3) showed a DeepLoc prediction of mitochondrion. Together, the GO CC terms and DeepLoc predictions in each set of hyperLOPIT compartment classifications indicate a high level of agreement of the TAGM-MAP classifications with alternative localisation approaches.

TAGM-MAP analysis provided comprehensive insight into the spatial organisation of the trypanosome cell with the classification of thousands of proteins to subcellular compartments. In some cases, proteins were not classified with sufficient confidence to a single compartment due to uncertainty attributed to dynamic protein localisation – localisation to more than one subcellular compartment. To provide insight into such cases, TAGM-MCMC (Markov-chain Monte-Carlo) was employed (Supplementary Data 10). 84-86% of the TAGM-MAP classifications match those of the TAGM-MCMC allocations. The key utility of TAGM-MCMC for these datasets is insight into the localisation of proteins that were unknown in TAGM-MAP due to potential dynamic localisation. Several cases highlight proteins that may exhibit localisation to more than one compartment. For example, in *T. brucei* PCF, where the mitochondrial membranes were particularly well resolved, MICU1 (mitochondrial calcium uptake 1) shows levels of uncertainty associated with the mitochondrial IM and OM. This protein functions in calcium import in the mitochondrion and potentially forms a contact site between the IM and OM (Fig. 3C)^10,49,50^. Similarly, succinate dehydrogenase, SDH1 shows uncertainty with mitochondrial matrix 2 and IM, this is supported by its localisation to the mitochondrial matrix and further functional association with SDH at the inner mitochondrial membrane (Fig. 3C)^51^.

### Trypanosome spatial proteomes demonstrate comprehensive and definitive resolution of subcellular niches

The four spatial proteomes were developed and characterised through the implementation of unsupervised and semi-supervised machine learning via the algorithms HDBSCAN, Novelty TAGM, TAGM-MAP and TAGM-MCMC. The TAGM-MAP classifications are the primary classifications and form the basis for the spatial proteomes reported in this work. The four datasets provide evidence of expression of ~5500 proteins with 2504–2795 classifications to 19–23 compartments per cell-type. Interrogation of the spatial proteomes, as visualised in Figs. 2–4 and Supplementary Figs. 2–6, demonstrate that known compartments of the cell were definitively resolved by the methodology employed here. All the major subcellular niches of the cell were represented in this analysis including soluble cytosolic proteins, large molecular complexes, and those in membranous compartments or cytoskeletal structures. Each of these datasets provide a spatial map that can be utilised separately, or in various combinations, to inform on individual protein localisations or protein components of cellular structures or organelles. In addition to the datasets included here, the data is also available in an interactive format via a R Shiny application.

The cytosol was one of the largest compartments. Some cytosolic protein complexes were separately resolved including the proteasome and proteasome regulatory particle, in addition to ribonucleoproteins 1 and 2 which were predominantly comprised of ribosomal proteins or translation initiation factors. Small membranous organelles found in the cytoplasm were also represented including the acidocalcisomes and glycosomes.

The nucleus was resolved into two compartments: the first contained chromatin components, while the second contained nucleolar and non-chromatin components. The latter was enriched with nucleolar proteins, though also included proteins involved in other nuclear-localised processes, such as splicing. There are examples of proteins expected to be nuclear classified to the cytosol. In some cases, this relates to trafficking, for example, several importins were classified to the cytosol. There are also cases where this may be due to rupture of or leakage from the nucleus during the lysis or separation method. The nucleus is particularly sensitive to rupture and the lysis method chosen here, which aims to generate a lysate enriched with all subcellular compartments, is not that which would be used for a nucleus-specific study.

The mitochondrion was particularly well resolved in this analysis including the matrix and membrane mitochondrial proteins in all cell-types. Further, in the PCFs, the mitochondrial IM and OM were resolved. Despite the lack of unsupervised clusters representative of the OM in BSFs, visual inspection indicated resolution of related proteins and the OM was designated as a compartment for classifications in all cell-types. Functional resolution of the mitochondrial matrix was observed as up to three distinct niches. The sub-grouping was not consistent between all cell-types, with some differences between matrix 1-3 in markers and classifications. Mitochondrial matrix 1 contains large and/ or small mitochondrial ribosomal proteins, with large ribosomal proteins in mitochondrial matrix 2 in *T. congolense* PCF and matrix 3 in BSF. Mitochondrial matrix 2 comprises proteins involved in diverse mitochondrial localised biochemical processes including RNA editing; iron-sulfur cluster assembly; and protein folding^52^. Mitochondrial matrix 3 was separated in *T. congolense* BSF only and enriched with a subset of large ribosomal proteins. This level of sub-organelle resolution is unprecedented for LOPIT studies. Further, when considered collectively as a single compartment (matrix and membrane proteins) there were 751–1091 proteins in the mitochondrial set, making it the largest compartment in this analysis (Table 1 and Supplementary Data 7).

Cytoskeletal compartments were represented by the flagellum 1 and 2, and microtubule structures 1 and 2. Flagellum 1 comprises the core cytoskeletal components of the flagellum including the axoneme and paraflagellar rod. In *T. congolense* BSF only, a subset of flagellar proteins was resolved in a second compartment designated flagellum 2 predominantly containing paraflagellar rod proteins. This resolution may be due to the flagellum-mediated adherence of *T. congolense* BSF to culture flasks affecting compartment integrity during harvesting^53,54^. Microtubule structures 1 comprises cytoskeletal proteins associated with flagellar and microtubule proximal structures including proteins localising to the bilobe, microtubule quartet, or basal body. Microtubule structures 2 comprises cytoskeleton interacting proteins, including those involved in cytoskeleton organisation, microtubule-based movement and cell division. Between the different cell-types this set includes proteins associated with the subpellicular microtubules, FAZ (flagellum attachment zone) filament and FAZ tip^55^. Many proteins in this set have been reported to localise to the cleavage furrow, function in cytokinesis, or interact with cytokinesis regulators^56–58^.

Components of the secretory pathway, including the ER, were also represented. The ER contains a high proportion (51–72%) of proteins predicted to contain TM domains, consistent with the presence of ER membrane proteins and proteins destined for the surface or other compartments. The collective post-ER secretory pathway, surface, endocytic pathway and endosomal pathway were resolved into up to three compartments named secretory/ endocytic 1–3. Secretory/ endocytic 1 includes Golgi, flagellar pocket, trafficking and lysosomal proteins. Secretory/endocytic 2 includes putative surface transporters including members of well-conserved gene families: amino acid (hOG00078), nucleoside (hOG00116) and hexose transporters (hOG00081)^59^. Secretory/endocytic 3 includes putative surface and endosomal proteins, as well as many proteins that are stage-specific or lack orthologs in the other species. Novelty TAGM analysis and review of related proteins in *T. congolense* PCF did not indicate there was a secretory/ endocytic 3 counterpart resolved here, so it was omitted for this cell-type.

### Comparative analysis highlights diversity between life stages of the same species

While the spatial proteomic outputs for the four cell-types can be utilised independently, the datasets can also be compared to inform on novel or divergent features between life-stages or species (Table 1 and Supplementary Data 7).

**Fig. 5:**
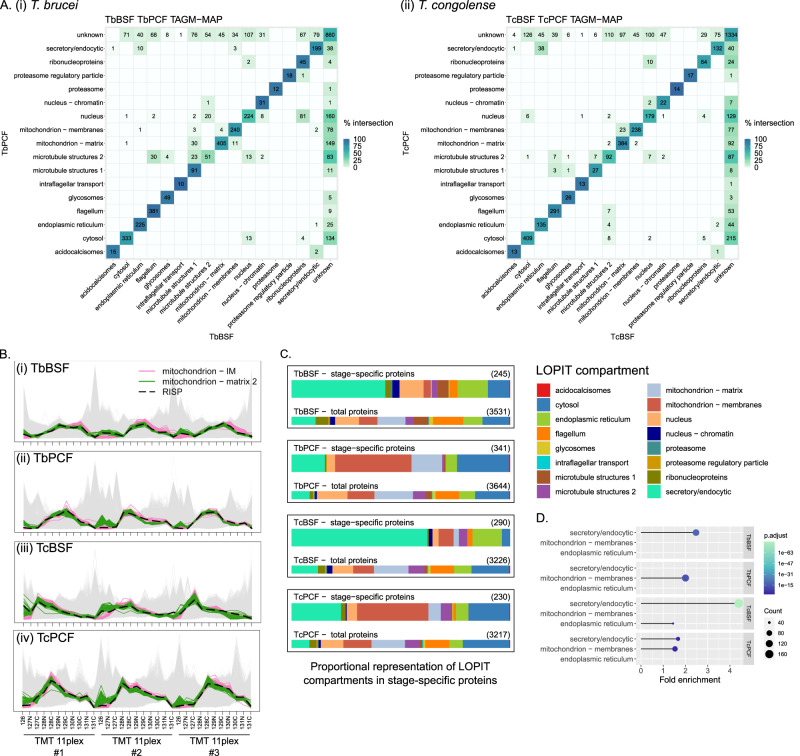
Comparative analysis of spatial proteomes between life-stages within species. **A** Heatmap of the agreement between compartments common proteins in (i) *T. brucei* BSF and PCF, and (ii) *T. congolense* BSF and PCF. Tile colour is scaled by the agreement between life-stages. **B** Abundance distribution profile for RISP in (i) *T. brucei* BSF, (ii) *T. brucei* PCF, (iii) *T. congolense* BSF and (iv) *T. congolense* PCF relating allocation to either mitochondrion – matrix in BSFs or IM in PCFs. **C** Proportional representation of compartments in stage-specific proteins visualised on bar-plots for indicated cell-type. **D** Lollipop chart showing the fold enrichment for over-represented compartments in stage-specific proteins for indicated cell-type. Circle size is scaled by count of proteins in indicated compartment and colour by p-value for the over-representation of compartment versus the background proteome. Enrichment analysis was performed using clusterProfiler (v4.0.5) ‘enricher’ function with default settings (hypergeometric test *p* < 0.05 with Benjamini and Hochberg adjustment).

To compare life-stages, common proteins between compartments of both the BSF and PCF within a species were visualised in a heatmap (Fig. 5A). 4844 and 5051 proteins were common to BSF and PCF in *T. brucei* and *T. congolense*, respectively. As expected, there was good agreement within each species in most compartments. The nucleus and microtubule structures showed the lowest level of agreement. Collectively, microtubule structures 2 has the least agreement between life-stages, with many of the differential localisations due to classifications to other cytoskeleton compartments (flagellum and microtubule structures 1) in the other life-stage. Given proteins involved in cell division with dynamic localisation are found in this set and that these cells were asynchronous, such differences will arise due to spatiotemporal divergences in the cell cycle between the BSFs and PCFs^60,61^. Proteins with differential localisation in the nucleus included 81 *T. brucei* proteins classified to the nucleus in PCF but ribonucleoproteins in BSF. This included markers for stress granules such as DHH1, MKT1, PABP2, PBP1 and SCD6^62^. *T. brucei* BSF was subject to longer harvesting than PCF as well as different wash conditions, which may have led to stress granule formation. Classification of these proteins to the nucleus in *T. brucei* PCF is likely associated with nuclear periphery granules which are reported to contain many of the same proteins as stress granules and be localised to the nuclear periphery^63^. There were also cases of classification to the nucleus in *T. brucei* BSF and the cytosol in PCF. As described earlier, leakage of the nucleus during the experimental method accounts for some of these in addition to proteins that traffic between the nucleus and cytosol.

Stage-specific differential localisation of proteins between other compartments was also observed, some of which may be due to mechanical differences resulting in differential dissociation during lysis. For example, the TAC (tripartite attachment complex) was not resolved as a single compartment and some TAC proteins classified to microtubule structures 1 in *T. brucei* BSF were in mitochondrial compartments in PCF. Given the position of the TAC at the basal body and mitochondrial membrane, such differential localisations may be the result of differential separation during lysis or diversity in the arrangement of these structures between cell-types. Other divergences appear to be bona fide, for example RISP (Rieske iron-sulfur protein), was classified to the mitochondrial IM in PCF *T. brucei* and *T. congolense*, but to the matrix in *T. brucei* BSF (Fig. 5B). It is also allocated to the matrix in *T. congolense* BSF albeit below the confidence threshold for classification. RISP is an essential subunit for the activity of complex III in the electron transport chain at the IM. Assembly of complex III occurs sequentially after import and so absence of one subunit may impair assembly. *T. brucei* BSF is reported to have a reduced electron transport chain with components of complex III and IV functionally absent^64^. Here, complex III subunit ubiquinol-cytochrome C reductase (Tb927.10.4280/ TcIL3000.A.H_000752100) was classified to the IM in both PCF stages but absent in both BSFs. In yeast, this protein precedes RISP in the assembly of complex III^65^. Hence, the absence of this protein is consistent with the presence of RISP in the matrix and not at the IM integrated with complex III.

There were 593-743 stage-specific proteins within each species – proteins exclusively present in only one life-stage per species. Representation of LOPIT compartments among stage-specific proteins was visualised on bar charts (Fig. 5C). Enrichment analysis indicated that the secretory/ endocytic compartment was enriched in proteins unique to *T. brucei* BSF, *T. congolense* BSF and *T. congolense* PCF (Fig. 5D). While the mitochondrial membranes were enriched in proteins unique to the PCF stages exclusively (Fig. 5D). Stage-specific secretory/ endocytic proteins in the BSFs includes proteins annotated as VSGs (variant surface glycoproteins) and ISGs (invariant surface glycoproteins), in addition to proteins implicated in host interaction, such as FHR (factor-H receptor) and HpHbR (haptoglobin-haemoglobin receptor) in *T. brucei* BSF^66,67^. In the PCFs, stage-specific secretory/ endocytic proteins include trans-sialidases, adenylate and guanylate cyclases and amino acid transporters. Stage-specific mitochondrial membrane proteins in PCFs include components of the electron transport chain such as cytochrome oxidase subunits. These data demonstrate the extent to which the secretory/endocytic compartments act as a point of developmental adaptation in both life-stages and the mitochondrion exclusively in PCFs.

### Comparative analysis highlights key regions of diversity between species

**Fig. 6:**
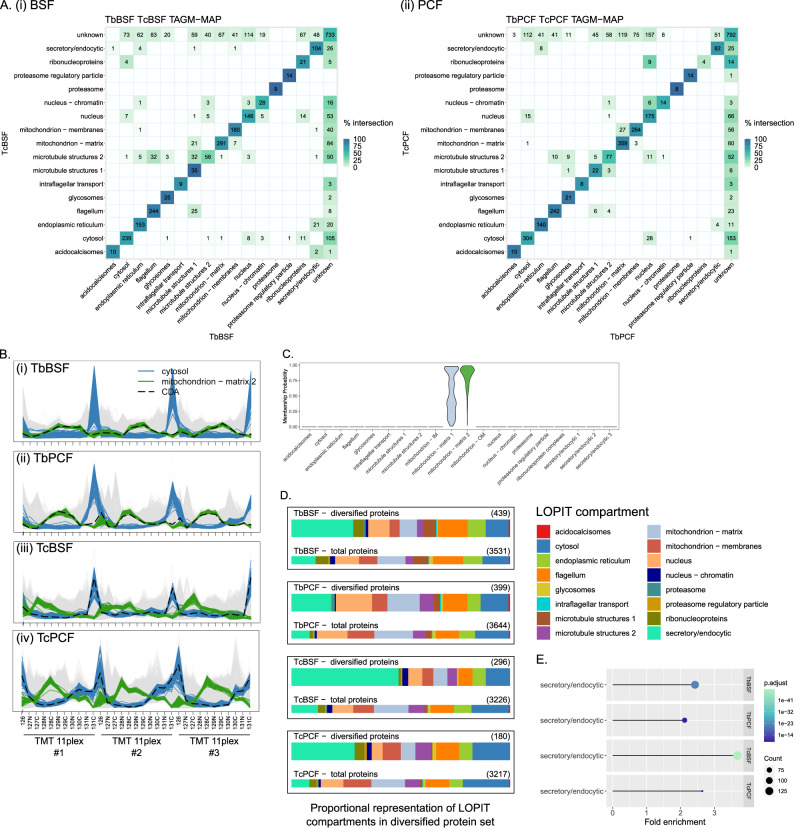
Comparative analysis of spatial proteomes between the same life-stage in each species. **A** Heatmap of the agreement between compartments in 1-to-1 orthologs of common proteins in (i) *T. brucei* BSF and *T. congolense* BSF, and (ii) *T. brucei* PCF and *T. congolense* PCF. Tile colour is scaled by the agreement between life-stages. **B** Abundance distribution profile for CDA in (i) *T. brucei* BSF, (ii) *T. brucei* PCF, (iii) *T. congolense* BSF and (iv) *T. congolense* PCF relating allocation to either mitochondrion in *T. brucei* or cytosol in *T. congolense*. **C** Violin plot showing distribution of localisation probabilities across each subcellular compartment according to TAGM-MCMC analysis in *T. brucei* PCF for CDA. **D** Proportional representation of compartments in diverse gene set proteins visualised on bar-plots for indicated cell-type. **E** Lollipop chart showing the fold enrichment for over-represented compartments in diverse gene set proteins for indicated cell-type. Circle size is scaled by count of proteins in indicated compartment and colour by *p*-value for the over-representation of compartment versus the background proteome. Enrichment analysis was performed using clusterProfiler (v4.0.5) ‘enricher’ function with default settings (hypergeometric test *p* < 0.05 with Benjamini and Hochberg adjustment).

To examine differences between species, the compartments of 1-to-1 orthologs, determined with OrthoFinder, between equivalent life-stages in each species were visualised in a heatmap (Supplementary Data 11 and Fig. 6A). Based on the 1-to-1 orthologs, there were 3635 proteins common to the BSFs and 3887 proteins to PCFs of *T. brucei* and *T. congolense*. Like the intra-species analysis, there was good agreement between species in most compartments. The compartments with the lowest level of agreement were again the nucleus and microtubules 2. A similar profile of divergence between compartments was observed as in the life-stage comparative analysis, described earlier. However, novel divergences also emerged, for example, CDA (cytidine deaminase) was classified to the cytosol in *T. congolense* BSF and PCF, but to the mitochondrial matrix in *T. brucei* BSF (Fig. 6B). It was also allocated to the mitochondrial matrix in *T. brucei* PCF, albeit below the TAGM-MAP confidence threshold and TAGM-MCMC analysis suggests this is due to uncertainty between mitochondrial matrix 1 and 2 (Fig. 6C and Supplementary Data 10). CDA has been localised to the mitochondrion in *T. brucei* PCF and functions in pyrimidine salvage with downstream steps occurring in the cytosol^68^. Localisation to the cytosol in *T. congolense* is also consistent with other eukaryotes, for example human CDA (P32320) is annotated as cytosolic^69^.

Next, proteins that did not display 1-to-1 orthology between *T. brucei* and *T. congolense* were assessed; these are cases where orthogroups contain more than one gene or an orthogroup is species-specific (within *T. brucei*, *T. congolense*, *T. vivax* and *T. cruzi*). Such cases are of particular interest when considering speciation and include expanded gene families as well as species-specific genome gains. To consider such genes with respect to the spatial proteomes, a set of proteins encoded by genes expanded or species-specific, according to the OrthoFinder analysis, was defined as the ‘diversified protein set’. 429–716 members of this set were present in each cell-type. Enrichment analysis indicated that the secretory/ endocytic compartment was enriched in diversified proteins for all cell-types (Fig. 6D, E). This set includes an orthogroup (hOG00009) with a single gene annotated as an atypical VSG in *T. brucei* expanded to 212 genes in *T. congolense*. While this protein is absent in the *T. brucei* proteomes, 14 members of this orthogroup were present in the *T. congolense* proteomes: 12 in BSF and four in PCF. Of the BSF proteins eight were classified to secretory/ endocytic 3; four are predicted to contain a GPI anchor and four are not – reminiscent of the GPI format of heterodimeric TfR (transferrin receptor) in which only one subunit possesses a GPI anchor^70^.

To further interpret the molecular processes of evolutionary diversification of these parasites, the ancestral origin of orthogroups and cases of gene expansion or contraction between *T. brucei*-*T. congolense* were examined. The secretory/ endocytic compartment was consistently enriched with orthogroups showing novelty and expansion in all cell-types, including for example none-many orthogroups between *T. brucei*-*T. congolense* and species-specific orthogroups (Supplementary Fig. 9A–C). 27 proteins in orthogroups specific to *T. brucei* or specific to *T. brucei* and *T. congolense* that were not annotated as VSGs or ISGs were uniquely present in *T. brucei* BSF in secretory/ endocytic 1-3. At least six are predicted to have a three-helical bundle like structure which has been observed in trypanosome surface proteins such as HpHbR and FHR (Supplementary Fig. 9D)^67,71–73^. This demonstrates the utility of this dataset, particularly when combined with other proteome-wide resources, in expediting studies focused on subcellular compartments of interest, such as the surface. Further, collectively this analysis highlights the secretory/ endocytic compartments as active subcellular niches of evolutionary diversification in these species.

## Discussion

In this study, the proteomes of two related but distinct African trypanosome species have been mapped for two life-stages. Collectively 5439–5731 proteins were detected and 2504–2795 classified to one of 19–23 subcellular compartments. A series of machine learning methods were implemented to yield granular datasets reflecting the intrinsic resolution within each cell type. The fidelity of the classifications was substantiated by orthogonal and computational approaches.

These comprehensive datasets comprise the highest number of proteins detected in a proteomic study in *T. congolense* to-date and among the highest for *T. brucei*. They are exemplary resources for the parasite research community and can be easily leveraged through integration with existing and future omics datasets. In addition to localisation information on thousands of proteins per cell-type, such proteome coverage has utility as evidence of protein expression in each cell-type. This is of particular value for *T. congolense* which has been subject to considerably fewer experimental studies than *T. brucei*, from studies of individual protein function through to proteome-wide analyses. As a particularly relevant example, *T. brucei* has been the subject of a proteome-wide localisation study that employs protein tagging and microscopy^9^. The capacity to conduct such a study is facilitated by the ability to readily genetically modify *T. brucei* PCFs, but not one that could be replicated for *T. congolense* at this time. The *T. congolense* analysis reported here has application in high-throughput protein curation. For example, approximately 800 proteins classified to subcellular compartments in *T. congolense* contain no curated GO CC annotation based on *T. brucei* 1-to-1 orthologs. In addition, thousands of genes in *T. congolense* are annotated as pseudogenes. Approximately 1200 of these were detected in the BSF or PCF spatial proteome indicating mis-annotation as pseudogenes and enabling further genome curation correction. These datasets also give insight into the proteomic consequence of genomic diversity between *T. brucei* and *T. congolense*. For example, there are many expanded orthogroups in *T. congolense*, however whether this translated to protein-level expansion was previously unknown for most. Here, several cases are evident where gene family expansion is reflected on the protein level with multiple proteins of the same orthogroup detected in *T. congolense*.

The hyperLOPIT datasets are particularly powerful when studied comparatively. The subcellular compartments involved in parasitic host adaptations and speciation are revealed through analysis of stage-specific proteins or those encoded by genes that were diverse between species, respectively. The stage-specific set was enriched with the mitochondrion exclusively in PCFs, with components of the electron transport chain represented for example. This strongly reflects the metabolic differences between life-stages in *T. brucei* and suggests similar differences exists within *T. congolense*^64,74^.

This work also highlights the key role of surface-related compartments in African trypanosome diversity. The collective post-ER secretory/ endocytic compartment was revealed as the major driver in stage-specific differences and genomic divergences in both *T. brucei* and *T. congolense*. The surface proteome represents the physical interface between the host and these parasites. Host-specific defence mechanisms and nutritional opportunities drive the evolution and selected expression of surface proteins and consequently surface proteomes differ considerably between life-stages. Distinct host ranges and distinct host microenvironments will also place differential selective pressure on the surface proteins expressed in each species. For example, unlike *T. brucei*, *T. congolense* BSFs adhere to erythrocytes and endothelial cells in vivo^53,54^. This not only necessitates the presence of biochemical features enabling adherence, but also impacts nutritional availability, and the stresses and host factors encountered within this microenvironment. The observation of enrichment of secretory/ endocytic proteins, which include surface proteins, in the stage-specific and diverse protein sets identifies the importance of these proteins in evolutionary adaptation to the distinct environments encountered by each cell type. Notably, these sets include numerous uncharacterised proteins and provide a promising repertoire of candidates for the molecular determinants of parasite diversity.

In summary, hyperLOPIT has been employed to spatially map the proteomes of *T. brucei* and *T. congolense*, each in the mammalian BSF and the insect midgut PCF. These highly comprehensive datasets not only provide rich expression and localisation information on thousands of proteins, but also reveal the key subcellular compartments involved in parasite diversity at a life-stage and species level.

## Methods

### Cell culture

*T. brucei* strain Lister 427 cells sourced from the laboratory of Mark Carrington (University of Cambridge) were used for BSF and PCF life-cycle stages. *T. brucei* BSF were maintained in HMI-9 10% FBS (fetal bovine serum) at 37 °C 5% CO_2_ 0.9 × 10^4^ to 1.96 × 10^6^ cells/ ml. *T. brucei* PCF were maintained in SDM-79 10% FBS at 27 °C 5% CO_2_ 2.5×10^5^ to 1.63×10^7^ cells/ ml. *T. congolense* strain IL3000 cells were used for BSF and PCF life cycle stages. *T. congolense* BSF cells were sourced from the laboratory of Catarina Gadelha (University of Nottingham), *T. congolense* PCF cells were sourced from the laboratory of Mark Carrington (University of Cambridge). *T. congolense* BSF were maintained in TcBSF-1 10% GS (goat serum) at 37 °C 5% CO_2_ at 1×10^5^ to 6.7×10^6^ cells/ ml^75^. *T. congolense* PCF were maintained in TcPCF-3 20% FBS at 27 °C 5% CO_2_ at 5 × 10^5^ to 1.6 × 10^7^ cells/ml^75^. For hyperLOPIT experiments logarithmically growing cells were scaled up in suspension in 75 cm^2^ culture flasks (T175s) for all cell types except *T. congolense* BSF, which were scaled up in adherent layers in 5-layer 875 cm^2^ flasks.

### Cell harvesting and lysis

Logarithmically growing cells were harvested and washed according the cell-type as follows: (i) *T. brucei* BSF were collected by repeated centrifugation (10 min 4635 x *g* 20 °C) with two-three centrifugation washes (10 min 1693 × *g* RT) in Voorheis PBS (136.9 mM NaCl, 3 mM KCl, 16 mM Na_2_HPO_4_, 3 mM KH_2_PO_4_, 45.9 mM sucrose, 10 mM glucose, pH 7.6) followed by up to wash in homogenisation medium (HM: 0.25 M sucrose, 10 mM HEPES, KOH pH 7.4, 1 mM EDTA) (ii) *T. brucei* PCF were collected by repeated centrifugation (10 min 4635 x *g* 20 °C) with three centrifugation washes (10 min 1693 × *g* RT) in serum-free SDM-79 followed two washes in HM, (iii) *T. congolense* BSF were washed once on-flask with serum-free TcBSF-1 followed by one-two on-flask washes with HM, collected by shaking into suspension and centrifugation, and followed by two centrifugation washes (10 min 1600 x *g* RT) in HM, and (iv) *T. congolense* PCF were collected by repeated centrifugation (10 min 4635 × *g* 20 °C) with three centrifugation washes (10 min 1600 × *g* RT) in serum-free TcPCF-3 followed two washes in HM. Each cell-type was ultimately suspended in chilled HM supplemented with protease inhibitors (cOmplete EDTA-free Proteinase inhibitor cocktail, Roche) at a density of 4 × 10^8^/ ml.

Cells were lysed by nitrogen cavitation (cell disruption vessel model 4639, Parr Instrument Company) in line with other work^28,34,76^. Briefly, the cell suspension was loaded into the pre-chilled vessel which was charged at 350–850 psi and incubated for 15 min before release of the lysate at 1–2 drops/second. The lysate was immediately brought to 2 mM magnesium acetate tetrahydrate and 500 U Benzonase (Merck) added, followed by 20 min incubation at RT and 10 min incubation on ice. Unlysed cells were removed by four centrifugations (5 min 200×*g* RT) to yield the clarified cell lysate. Further details are available in Supplementary Data 1.

### Subcellular fractionation

Subcellular fractionation was performed as previously described with some modifications^23,28^. First, the crude membranes were enriched with an iodixanol cushion whereby the cell lysate was underlaid with 6% and 25% iodixanol solutions in HM and centrifuged at 146,498 × *g* in SW40 Ti rotor (Beckman Coulter) or 100,095 × *g* in a SW55 Ti rotor (Beckman Coulter) at 4 °C for 1.5 h. An aliquot was collected from the top of the cushion and taken as the soluble fraction. The crude membranes were enriched in two visible bands at the interfaces between the iodixanol layers. These were collected using a Pasteur pipette, diluted with HM and pelleted by centrifugation at 200,309 × *g* in a SW55 Ti rotor (Beckman Coulter) at 4 °C for 1 h. The pelleted crude membranes were suspended in 32% iodixanol solution in HM with 10 strokes of a Dounce homogeniser (Pestle B 885300-0002, Kimble-Chase, clearing distance of 0.0127–0.0635 mm). The suspended crude membranes were then underlaid below a pre-formed iodixanol gradient comprised of sequentially underlaid steps of iodixanol solution in HM (20%, 23%. 26% and 29% iodixanol) which had been allowed to diffuse overnight at 4 °C. After underlaying, the gradient was centrifuged at 99,820 × *g* in a VTi65.1 rotor (Beckman Coulter) at 4 °C for 12 h. The gradient was collected in fractions by piercing the bottom followed by the neck and collecting the solution dropwise from the bottom into approximately 22 0.5 ml fractions. After collection the Ri (refractive index) was measured for each fraction using a refractometer (Bellingham Stanley) and protein content measured by Bradford assay. Fractions were either stored at −80 °C or immediately processed for protein extraction.

### Sample preparation for LC-MS/MS analysis

Protein was extracted from the soluble fraction by the addition of 4-5X the volume of ice-cold acetone. Protein was extracted from the iodixanol-containing gradient fractions by TCA (trichloroacetic acid) precipitation whereby fractions were adjusted to ~10–15% TCA to precipitate protein, followed by 10% TCA washes and acetone washes. During acetone washes, pellets were subjected to sonication (5 cycles of 30 s ON/ OFF at high power, Bioruptor Plus ultrasonic disintegrator, Diagenode). All samples were then solubilised in 100 mM TEAB (triethylammonium bicarbonate) buffer (pH 8.5) with 0.2% SDS (sodium dodecyl sulfate) 8 M urea and subjected to repeated sonication. Protein content was measured by BCA (bicinchoninic acid) assay. Fractions (0.2 or 0.4 µg loading) were analysed by Western blotting using standard methods with antibodies against a panel of compartment marker proteins. Antibodies (anti-ISG65, anti-MI.2 C D88 (VSG221), and anti-DHH1) were sourced from the laboratory of Mark Carrington (University of Cambridge). Antibodies (anti-BiP, anti-CatL and anti-mtHSP70) were sourced from the laboratory of James Bangs (University of Buffalo). Antibodies (anti-Histone H3 (ab1791), anti-rat IgG H&L (HRP) (ab97057) and anti-rabbit IgG H&L (HRP) (ab6721)) were sourced commercially from Abcam and anti-Tubulin [YL1/2] (ab6160/ MAB1864) was sourced from Abcam and Sigma-Aldrich respectively. Antibodies were used at approximately the following dilutions: anti-ISG65 (5000), anti-MI.2 C D88 (VSG221) (50000), anti-DHH1 (5000), anti-BiP (1500000), anti-CatL (5000-10000), anti-mtHSP70 (10000), anti-Histone H3 (1000-5000), anti-Tubulin [YL1/2] (1500-2000), anti-rat IgG H&L (HRP) (20000) and anti-rabbit IgG H&L (HRP) (30000). Note that in all cases, once diluted in blocking solution, antibodies were stored and re-used.

For each experiment, gradient fractions were concatenated to form 10 pools (30–98 µg). Fraction concatenation was guided by Western blot analysis to maximise subcellular compartment resolution in individual runs while varying resolution between runs where possible, with the constraint of protein availability. An 11th sample was formed with the soluble fraction. Samples were reduced, alkylated and trypsinised as per Barylyuk et al. (2020). After trypsination, peptides were quantified for all runs except for *T. brucei* BSF iteration #1 (Pierce Quantitative Fluorometric Peptide Assay) and peptide quantity normalised for all fraction pools. Peptides (20-56 µg) were labelled with TMT-11plex tags (A37725 or 90111 supplemented with A37724, Thermo Fisher Scientific). Briefly, 0.8 mg of tags equilibrated to room temperature were suspended in acetonitrile (41-90 µl) and added (20.5-41 µl) to peptide mixture (54- 115 µl) creating conditions at 1X or 0.5X the TMT amount as per the manufacturer instructions. The reaction was allowed to proceed for 2 h at 25 °C with agitation (800 rpm, Eppendorf ThermoMixer) before the addition of 8 µl 5% (v/v) hydroxylamine with incubation for 1 h at 25 °C with agitation (800 rpm). The TMT-tagged samples were then pooled and reduced to dryness by refrigerated vacuum centrifugation (Labconco).

The TMT pools were desalted with modification from Mulvey et al. (2017) using Sep-Pak tC18 Plus Light Cartridge (WAT036805, Waters). Briefly, using a syringe, cartridges were conditioned with 2 ml acetonitrile, 2 ml elution buffer (70% acetonitrile, 0.05% acetic acid), 2 ml desalting buffer (0.05% acetic acid) and 4 ml loading buffer (0.1% TFA (trifluoroacetic acid)). Peptides (suspended in 0.1% TFA adjusted to approximately pH 2) were loaded onto the cartridges followed by 4 ml loading buffer and 4 ml desalting buffer. Peptides were eluted in 1.6 ml elution buffer and reduced to dryness by refrigerated vacuum centrifugation. Desalted peptides were fractionated by high-pH reverse phase chromatography as per Mulvey et al. (2017) using a Waters Acquity UPLC system with a BEH C18 (1.7 μm, 2.1 × 150 mm) column and BEH C18 (1.7 μM, 2.1 × 5 mm) VanGuard pre-column^23^.

### LC-MS/MS analysis

All MS runs were performed on an Orbitrap Fusion^TM^ Lumos^TM^ Tribrid^TM^ instrument coupled to a Dionex Ultimate^TM^ 3000 RSLCnano system (Thermo Fisher Scientific). UPLC fractions were concatenated to 17-19 pools and each resuspended in 20 μL of 0.1% (v/v) formic acid and approximately 1 μg of peptides was loaded per injection for LC-MS/MS analysis. The nano-flow liquid chromatography method for LC-MS/MS analysis was set as per previous work^23^. The MS workflow parameters with XCalibur (v3.0.63) were also as per previous work with the exception of the Orbitrap resolution which was instead set to 50,000^23^.

### Proteomic data quantification and processing

Peptide identification and quantification was performed in ProteomeDiscoverer (v2.4) with Mascot (v2.7.0, Matrix Science) using sequence databases from TriTrypDB (v50) according to species: *T. brucei* (TREU927 with Lister 427 BES40 genes appended), *T. congolense* (IL3000 2019). Contaminant databases included (i) an expanded GPM (Global Proteome Machine) cRAP (common Repository of Adventitious Proteins) from CamProtR (v0.0.0.9000, github.com/CambridgeCentreForProteomics/camprotR) with Benzonase (P13717^69^) appended, and (ii) the cRFP (common Repository of FBS Proteins) database^77^. Precursor error and mass fragment tolerances were set to 10 ppm 0.6 Da respectively, reporter ions were quantified using the most confident centroid method, and trypsin was set as the enzyme of choice with a maximum of 2 missed cleavages. Static modifications included: Carbamidomethyl (C), TMT6plex (N-term) and TMT6plex (K). Dynamic modifications included: Deamidated (NQ), Oxidation (M), TMT6plex (S) and TMT6plex (T). Percolator was used to assess the FDR (false discovery rate) and only high-confidence peptides were retained. PSM level data was exported, and identifiers were converted to gene identifiers based on the fasta header. The data was further processed in R with following parameters: Number of Protein Groups = 1; Rank = 1; Search Engine Rank = 1; Intensity > 1e3; Average Reporter S/N > = 5; Isolation Interference <= 50%^32^. PSMs matching to contaminants (cRAP and cRFP) and those with missing values in any of the 11-plex TMT quantitation channels were removed. PSM intensities were sum-normalised then median-aggregated to the protein level. Each 11-plex TMT experiment was then concatenated to form a 33-plex dataset and proteins with missing values in any of the experiments were removed^35^.

### Computational methods for spatial proteome characterisation

Data inspection and analysis was performed in R (v4.1.0) predominantly using Bioconductor packages MSnbase (v2.18.0), pRoloc (v1.32.0), tidyverse (v1.3.1), clusterProfiler (v4.0.5) and ggplot2 (v3.3.3)^25–27,78–82^. Normalised 33-plex hyperLOPIT datasets were imported and subjected to dimensionality reduction via t-SNE as per Barylyuk et al. (2020)^28,38^. Unsupervised clustering was performed with the algorithm HDBSCAN using the Python (v3.7.1) library hdbscan (v0.8.19) on normalised hyperLOPIT datasets^40,83^. The default parameters were used except the following: minimum samples = 8; minimum cluster size = 9; cluster selection method = ‘leaf’; generate minimum spanning tree = TRUE.

For the development of *T. brucei* markers, an initial marker protein set was prepared by manual literature review and GO CC annotation guided by HDBSCAN clustering information and visual inspection of protein abundance distribution profiles. Automated literature review for localisation evidence of HDBSCAN cluster members was also performed using the ScienceDirect API (https://www.elsevier.com/solutions/sciencedirect/librarian-resource-center/api) with the Python (v3.7.1) library elsapy (v0.5.0). Proteins that appeared to be outliers or movers between life-stages were excluded and the same markers were used for *T. brucei* BSF and PCF. For the development of the *T. congolense* markers, an initial marker set was prepared by taking the orthologs of the *T. brucei* markers or TAGM-MAP allocations (the most probable compartment localisation of each protein as performed with TAGM-MAP – see below) that had supporting localisation evidence in *T. brucei*. These were then further selected guided by HDBSCAN clustering and visual inspection of protein abundance distribution profiles. This initial marker set was used for Novelty-TAGM semi-supervised analysis to allow for the discovery of any unlabelled compartments as phenotypes, in addition to allocation of proteins to defined compartments^46^. Novelty TAGM parameters were set to defaults and the algorithm was run for 10,000 iterations, 5000 were discarded automatically, thinning was set to 10 and 9 independent chains were run. The results of the Novelty-TAGM analysis were inspected for new phenotypes representing resolved subcellular niches and expansions in existing compartments for additional candidate markers based on localisation evidence in *T. brucei* orthologs. Due to differential resolution of several compartments between BSF and PCF, the same markers but with differential merging/ partitioning of sub-compartments were used in *T. congolense*.

Protein localisation predictions were generated based on the TAGM model^26,33^. TAGM-MAP classifications of the 33-plex datasets are the primary classifications and form the basis for the spatial proteomes reported in this work. TAGM-MAP model parameters were generated using the default settings to determine the posterior allocation and outlier probabilities of each protein. Each non-marker protein was allocated to a compartment representing the most probable localisation of all the compartments. The localisation probability was computed as the product of the allocation probability and the complement of the outlier probability ($P({localisation})=P({allocation})\times (1-P({outlier}))$). Classifications were then generated by applying two thresholds to these allocations: localisation probability >0.999 and separately an outlier probability <5 E-5. Proteins that did not meet the thresholding criteria were designated as ‘unknown’. To assess the reproducibility of classifications produced by individual experimental iterations, TAGM-MAP was also performed with the default settings on each 11-plex dataset separately. Classifications were retained if they exceeded a localisation probability >0.99. To assess the variability in classification between the experiments, datasets were compared pairwise using the adjusted Rand index which assigns a score of 0 if consistency is what is expected at random and 1 for perfect consistency using the R package mmclust (v1.0.1)^47^. To avoid inflating or deflating the ARI due to an excess of “unknown” allocations these were filtered before comparison. Analysis using TAGM-MCMC was then used to provide insight into proteins that were unknown according to TAGM-MAP where it could be due to dynamic protein localisation. This model was implemented using Markov-chain Monte-Carlo. The collapsed Gibbs sampler was run in parallel for 9 chains (*T. brucei*) and 4 chains (*T. congolense*) with each chain run for 10,000 iterations. Convergence was assessed using the Gelman-Rubin’s diagnostic and all Markov chains were retained for *T. congolense*; whilst for *T. brucei* the best two chains were retained. No thresholding criteria was applied with protein allocations and compartment joint probabilities are reported. Joint probabilities were used to evaluate proteins that may exhibit localisation to more than one compartment.

### Protein feature annotation and analysis

Protein feature annotation was predominantly taken from TriTrypDB (downloaded on 01/06/2021) for features including protein names, descriptions, molecular weights, TM domains, signal peptides, GO terms and Pfam descriptions^43^. GPI anchor presence was predicted using NetGPI online (v1.1, services.healthtech.dtu.dk/service.php?NetGPI) with sequences from TriTrypDB (v50) as used for all proteomic analysis^44^. Protein pI (isoelectric point) values were predicted with pIR (v0.99.0) using the Bjell method using sequences from TriTrypDB (v50) as used for all proteomic analysis^41,42^. DeepLoc (v1.0) subcellular localisation predictions were generated locally in Python (v3.7.1) using pre-computed model parameters as available online (https://services.healthtech.dtu.dk/software.php)^48^. Sequences for DeepLoc were obtained from TriTrypDB as follows: *T. brucei* TREU927 (downloaded 28/02/2021) with BES40 genes appended from those used for all proteomic analysis (v50); *T. congolense* IL3000 2019 (downloaded 01/03/2021).

Inference of orthologous groups (OG) between *T. brucei* (TREU927 supplemented with Lister 427 BES40 genes), *T. congolense* (IL3000 2019), *T. vivax* (Y486) and *T. cruzi* (CL Brener Esmeraldo-like) was performed using OrthoFinder 2.5.2 supplying a fixed species tree (Supplementary Fig. 9), and with option -y for splitting OGs into hierarchical OG (hOG) based on automated tree reconciliation^84^. All proteomes were obtained from TriTrypDB (v50)^43^. Similarity searches were performed using BLAST (option -S blast). Any species-specific genes that are commonly excluded from the OrthoFinder output were separately added as single-gene OGs. Furthermore, inspection of hOGs revealed oversplitting of a number of the original OGs, which where manually corrected (Supplementary Data 11). Hence corrected hOG identifiers were used as the index. Additional annotation was added (Supplementary Data 11): (i) phylogenetic profile: binary presence/absence profile of genes in a given hOG across each species, (ii) origin: the inferred hOG ancestral origin node (N0-N6, see Supplementary Fig. 9), (iii) the categorical representation (loss/ none/ one/ two/ many) of the gene count in a given hOG for each species (loss can only be inferred in cases where genes of at least two species are present in an hOG) and iv) the categorical representation (loss/ none/ one/ two/ many) of the gene count in a given hOG specifically for *T. brucei*-to-*T. congolense*. Fasta identifiers were converted to the gene identifiers based on the source TriTrypDB proteome fasta headers.

To define stage-specific proteins, lists of proteins unique to a given stage compared with the other stage of the same species were compiled and are referred to as such. The diversified protein set was developed based on encoding genes in species-specific and expanded orthogroups defined as follows: (i) species-specific: orthogroups only present in the indicated species, and (ii) expanded: orthogroups with >=2X the number of genes versus the counterpart in *T. brucei* or *T. congolense* accordingly. Cases of two-one gene count orthogroups in *T. brucei*-*T. congolense* where two fasta header identifiers matched to a single gene identifier were removed from this set in *T. brucei*.

Enrichment analysis was performed using clusterProfiler (v4.0.5) ‘enricher’ function with default settings^82^ (hypergeometric test p < 0.05 with Benjamini and Hochberg adjustment) testing for: (i) enrichment of GO CC terms in either all proteins or non-marker proteins in each subcellular compartment against the background of the corresponding proteome, (ii) enrichment of LOPIT compartments in stage-specific proteins or diverse gene set against the background of the corresponding proteome and (iii) enrichment of hOG origin annotations in each LOPIT compartment set against the background of the corresponding proteome.

### Statistics and reproducibility

Each of the final 33-plex datasets per cell type are comprised of three concatenated 11-plex iterations of the experimental procedure and Bayesian statistics were then used to analyse the concatenated data. For further details see Supplementary Note. Proteins with a missing value in any of the 33 datapoints per cell type were excluded from further analysis. Protein features were examined according to unsupervised clusters, subcellular compartments, cell-type specificity and species diversity using enrichment analysis. No statistical methods were used to predetermine sample sizes. The experiments were not randomised. The Investigators were not blinded to allocation during experiments and outcome assessment.

## Supplementary information

Supplementary Information

Description of Supplementary Files

Supplementary Data 1

Supplementary Data 2

Supplementary Data 3

Supplementary Data 4

Supplementary Data 5

Supplementary Data 6

Supplementary Data 7

Supplementary Data 8

Supplementary Data 9

Supplementary Data 10

Supplementary Data 11

## Data Availability

The MS proteomics data generated in this study have been deposited to the ProteomeXchange Consortium via the PRIDE partner repository with the data identifier PXD035426^85^. Interactive versions of each dataset can be viewed online through R shiny applications at https://proteome.shinyapps.io/tbrucei_bsf/, https://proteome.shinyapps.io/tbrucei_pcf/, https://proteome.shinyapps.io/tcongolense_bsf/, and https://proteome.shinyapps.io/tcongolense_pcf/. Each spatial proteome is also integrated with TriTrypDB (https://tritrypdb.org/tritrypdb/app)^43^. Datasets are also available within the R package pRolocdata version 1.37.1. at https://bioconductor.org/packages/release/data/experiment/html/pRolocdata.html^25,86^. Further information and requests for resources and reagents should be directed to and will be fulfilled by the corresponding author, Paula MacGregor (paula.macgregor@bristol.ac.uk). Source data are provided with this paper.

## Source data

Source data
