# Supplementary material for: Mapping diversity in African trypanosomes using high resolution spatial proteomics
Source: Nat Commun. 2023 Jul 21;14:4401. doi: 10.1038/s41467-023-40125-z (PMC10361982; doi:10.1038/s41467-023-40125-z)
Supplement: Supplementary file 1 — Supplementary Information [file 41467_2023_40125_MOESM1_ESM.pdf]

Supplementary Information File

Contents

Supplementary Figures………………………………………………………………………………………………… 2 to 11

Supplementary Note…………………………………………………………………………………………………… 12

Supplementary References………………………………………………………………………………………….. 12 to 14

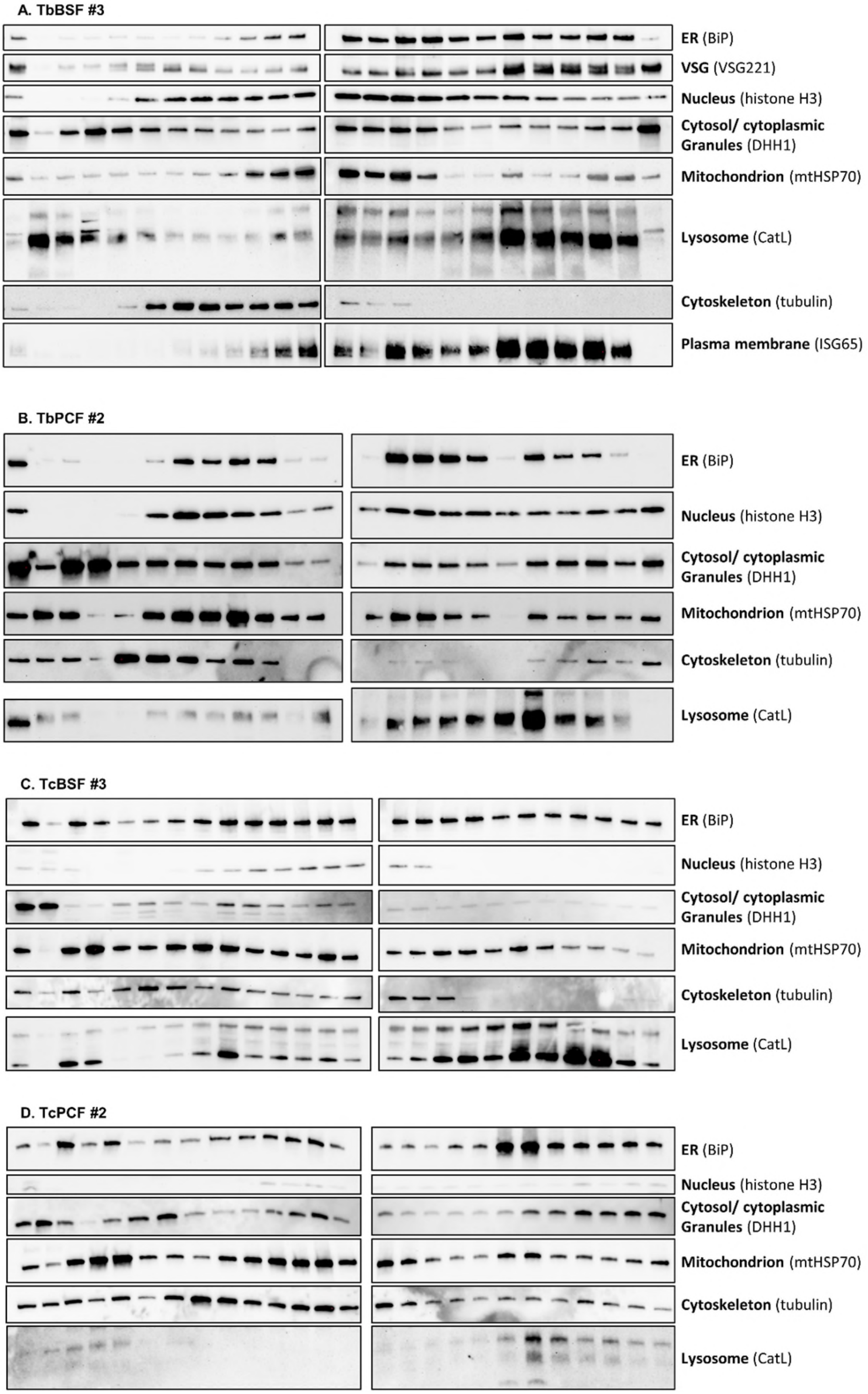

Supplementary Figure 1. Western blot analysis was used to crudely assess compartment resolution in fractions following equilibrium density gradient centrifugation. Exemplar western blots are shown, each of

which were loaded with 0.4 µg protein as follows: (A) *T. brucei* BSF iteration #3 of 3: Whole-cell lysate, density gradient fractions 1-22, soluble fraction, (B) *T. brucei* PCF iteration #2 of 3: Whole-cell lysate, density gradient fractions 1-21, soluble fraction, (B) *T. congolense* BSF iteration #3 of 3: Whole-cell lysate, soluble fraction, crude membrane fraction, density gradient fractions 1-23, and (D) *T. congolense* PCF iteration #2 of 3: Whole-cell lysate, soluble fraction, crude membrane fraction, density gradient fractions 1-23. Blots were probed sequentially or in combination using a panel of antibodies against the following compartments: ER (αBiP), nucleus (αhistone H3), cytosol/ cytoplasmic granules (αDHH1), mitochondrion (αmtHSP70), lysosome (αCatL), cytoskeleton (αtubulin), plasma membrane (αISG65). In addition, the VSG (αVSG221) was separately evaluated in *T. brucei* BSF. Each experiment was performed with three biological iterations with varying conditions, see Supplementary Note for more information. Uncropped images can be found in the Source Data File.

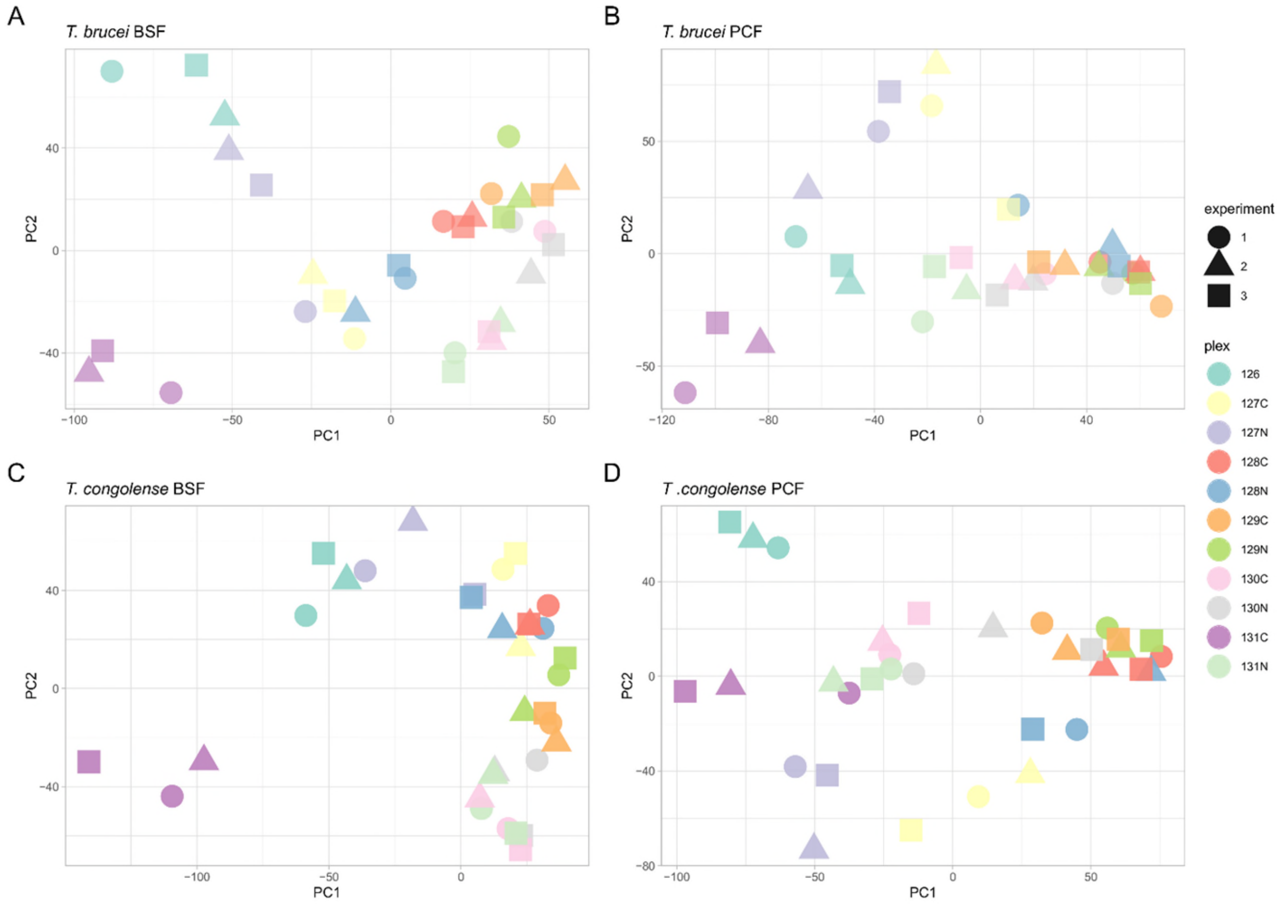

Supplementary Figure 2. Principal component analysis of the 11 fractions per individual experimental iteration for each cell type. A) *T. brucei* BSF, (B) *T. brucei* PCF, (C) *T. congolense* BSF, and (D) *T. congolense* PCF.

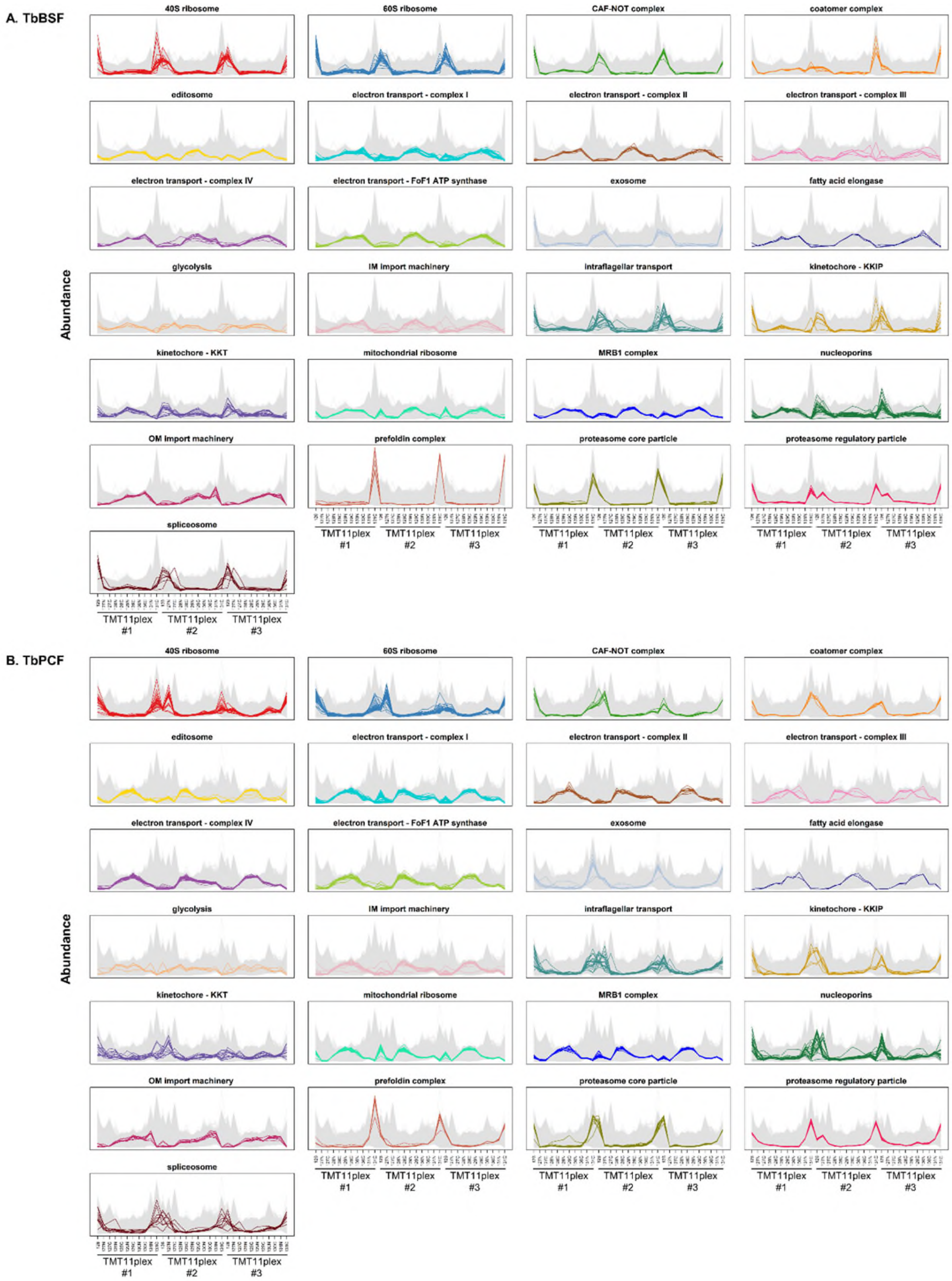

Supplementary Figure 3. Protein abundance distribution profiles of functionally related proteins (as per Supplementary Data 3) in (A) *T. brucei* BSF and (B) *T. brucei* PCF [1-24]. Normalised protein intensities (Abundance) are displayed on the Y-axis and TMT channels, corresponding to three concatenated 11-plex experiments (#1-3), on the X-axis.

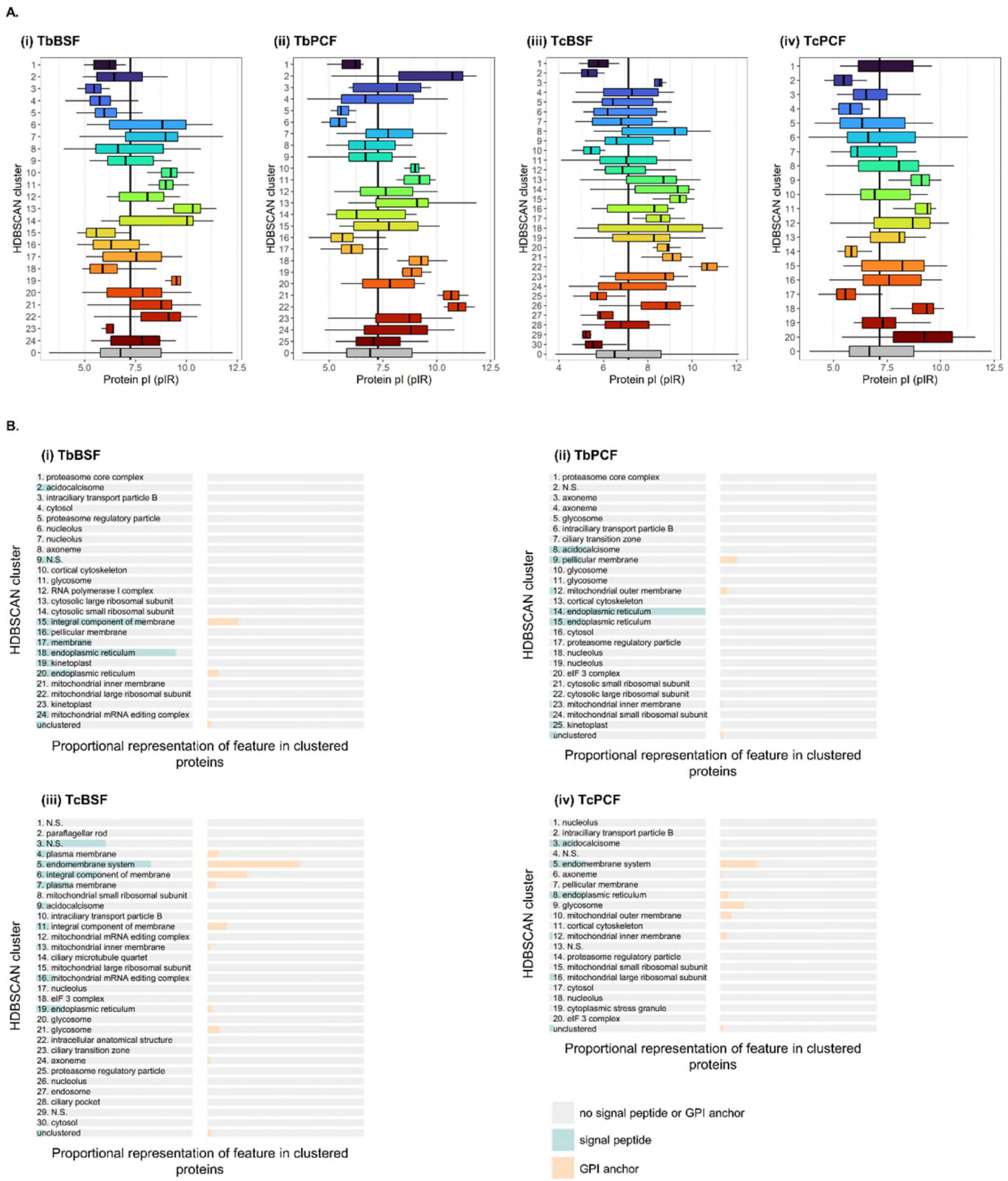

Supplementary Figure 4. Distribution of protein biochemical features in unsupervised HDBSCAN clusters. (A) Box plots showing distribution of computed pI values across each HDBSCAN cluster in i) *T. brucei* BSF, ii) *T. brucei* PCF, iii) *T. congolense* BSF, and iv) *T. congolense* PCF. n=5439, 5587, 5644 and 5731 proteins respectively where n= the total number of proteins examined per plot over each single 33-plex datatset per cell type. The centre line across each box plot represents the entire dataset mean. Within each cluster's individual box plot the centre line represents the subset data median, the box limits represent the first and third quartiles, and the whiskers extend to the largest and smallest values no further than 1.5X the interquartile range. (B) Bar plots of proportional representation of proteins predicted to contain signal peptides (green) or GPI anchors (orange) and significantly represented GO CC terms in each cluster in indicated cell-type (N.S. = not significant, with no significantly represented terms).

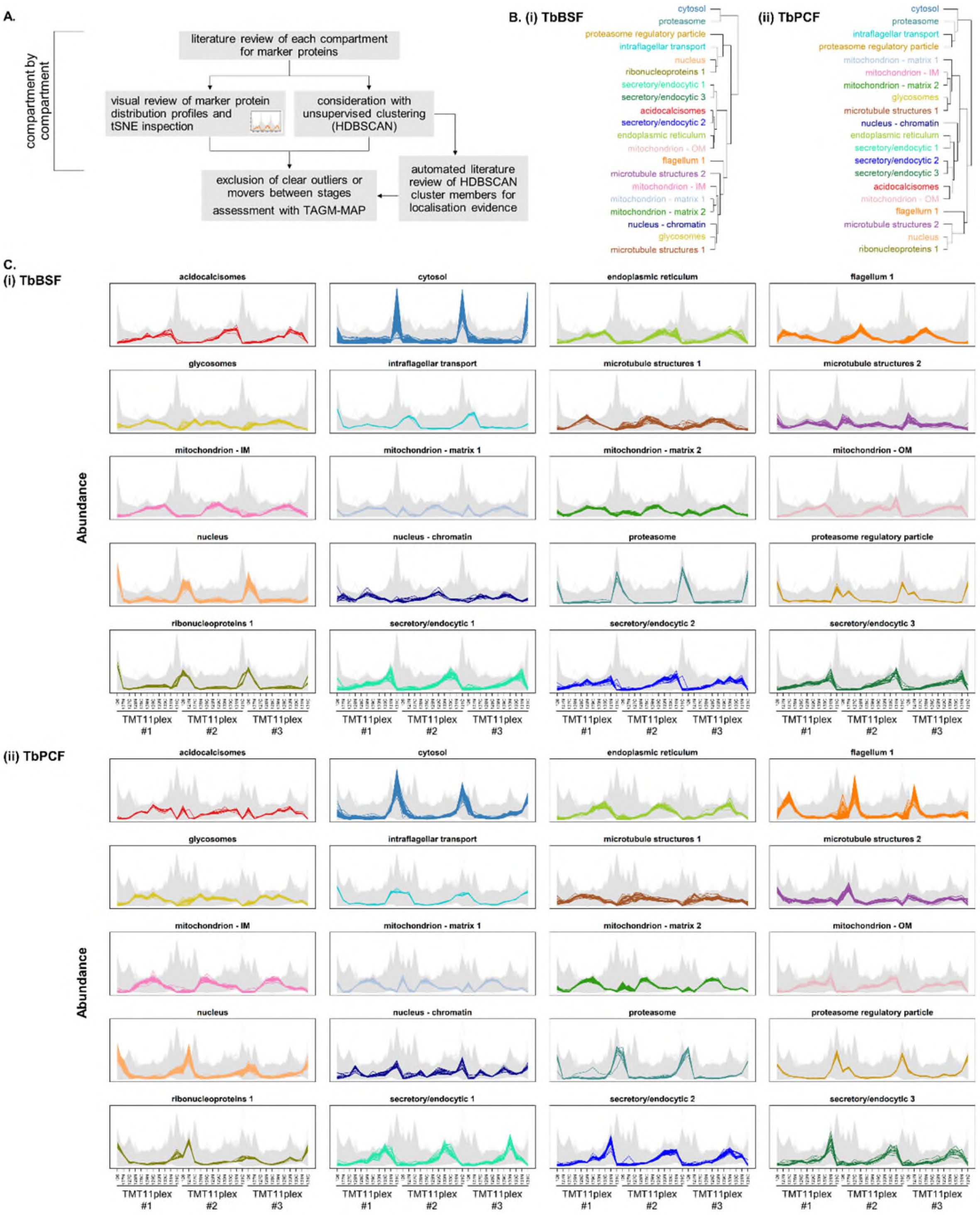

Supplementary Figure 5. *T. brucei* compartment marker proteins. (A) Schematic of the strategy used for the development of *T. brucei* compartment marker proteins. (B) Hierarchical representation of the similarity in average marker protein abundance profile for each compartment in dendrograms for i) *T. brucei* BSF and ii) *T. brucei* PCF. This indicated generally similar resolution of compartments across all cell-types. (C) Protein abundance distribution profiles of compartment marker proteins in i) *T. brucei* BSF and ii) *T. brucei* PCF. Normalised protein intensities (Abundance) are displayed on the Y-axis and TMT channels, corresponding to three concatenated 11-plex experiments (#1-3), on the X-axis.

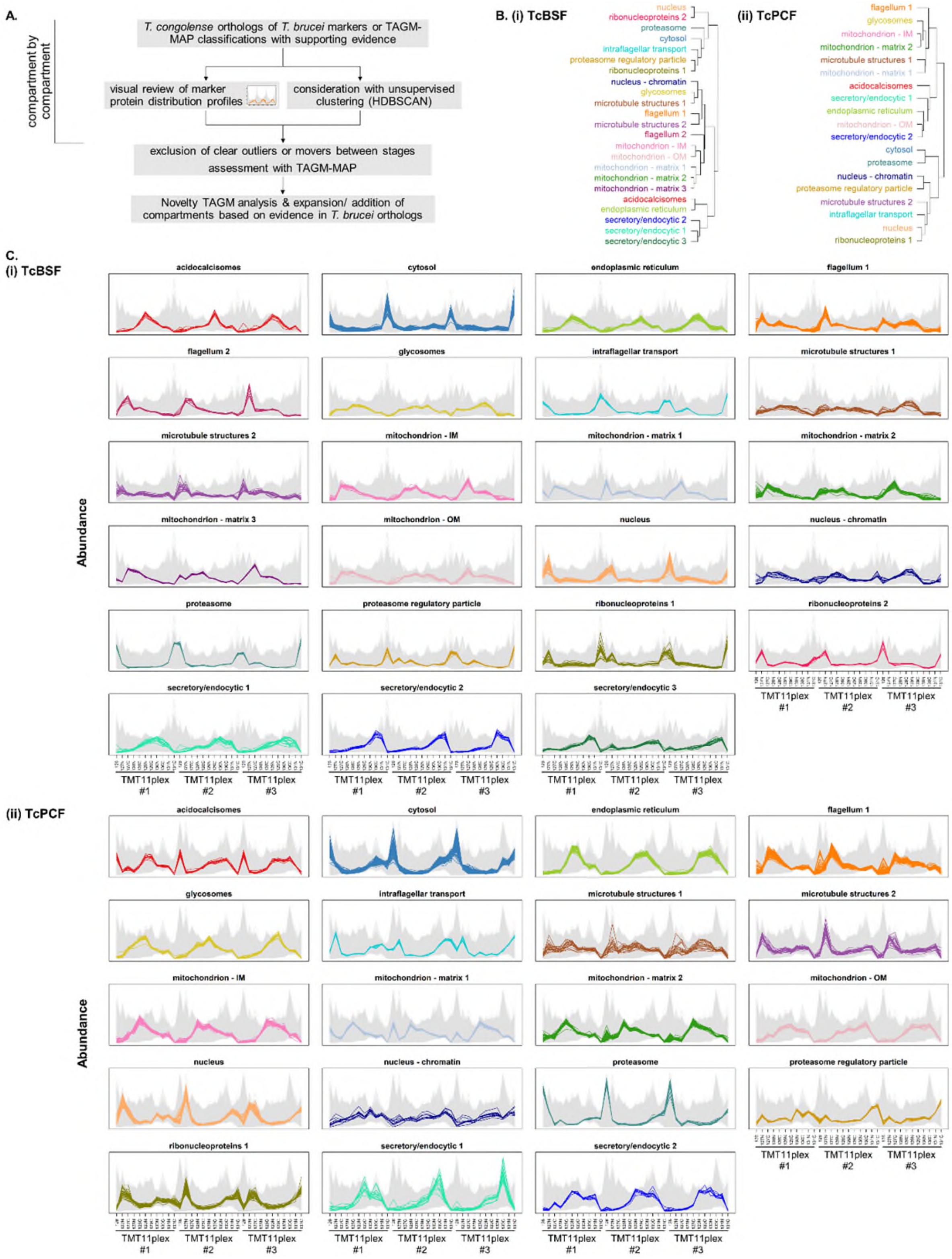

Supplementary Figure 6. *T. congolense* compartment marker proteins. (A) Schematic of the strategy used for the development of *T. congolense* compartment marker proteins. (B) Hierarchical representation of the similarity in average marker protein abundance profile for each compartment in dendrograms for i) *T. congolense* BSF, and ii) *T. congolense* PCF. This indicated generally similar resolution of compartments across all cell-types. (C) Protein abundance distribution profiles of compartment marker proteins in i) *T. congolense* BSF and ii) *T. congolense* PCF. Normalised protein intensities (Abundance) are displayed on the Y-axis and TMT channels, corresponding to three concatenated 11-plex experiments (#1-3), on the X-axis.

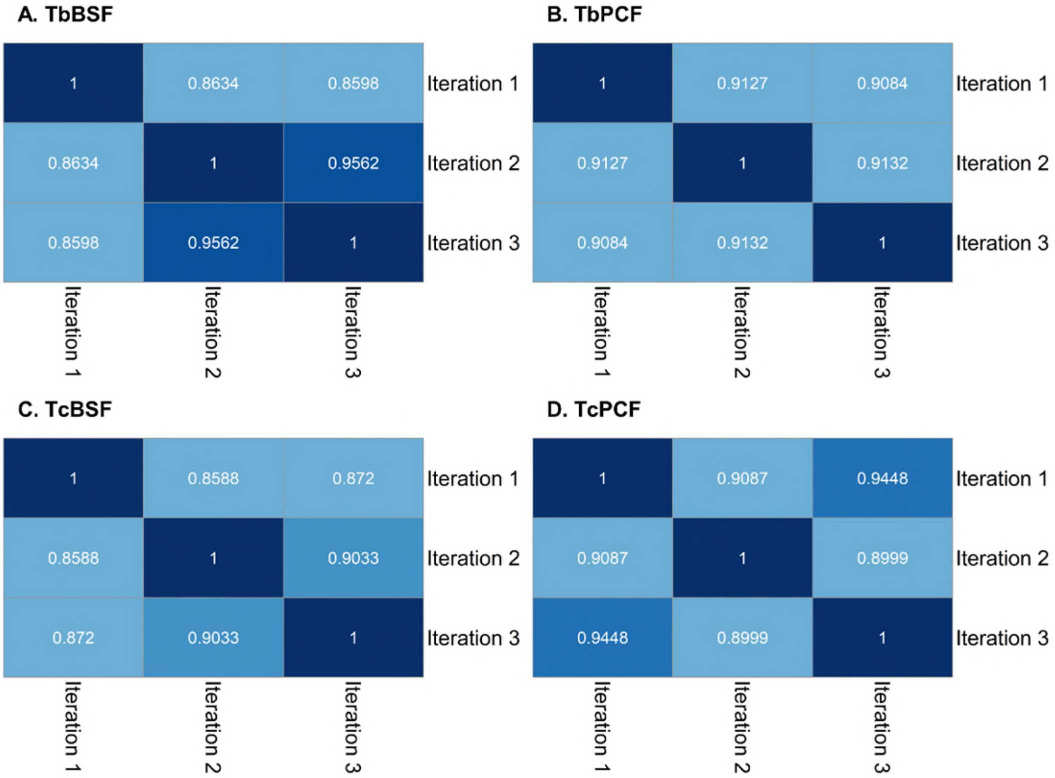

Supplementary Figure 7. Reproducibility analysis of TAGM-MAP classifications in individual experimental iterations for each cell-type. To assess the variability in classification between the experimental iterations, 11-plex datasets were compared pairwise using the adjusted Rand index in (A) *T. brucei* BSF, (B) *T. brucei* PCF, (C) *T. congolense* BSF, and (D) *T. congolense* PCF. The adjusted Rand index assigns a score of 0 if consistency is what is expected at random and 1 for perfect consistency[25]. Unclassified ("unknown") proteins were removed filtered before comparison.

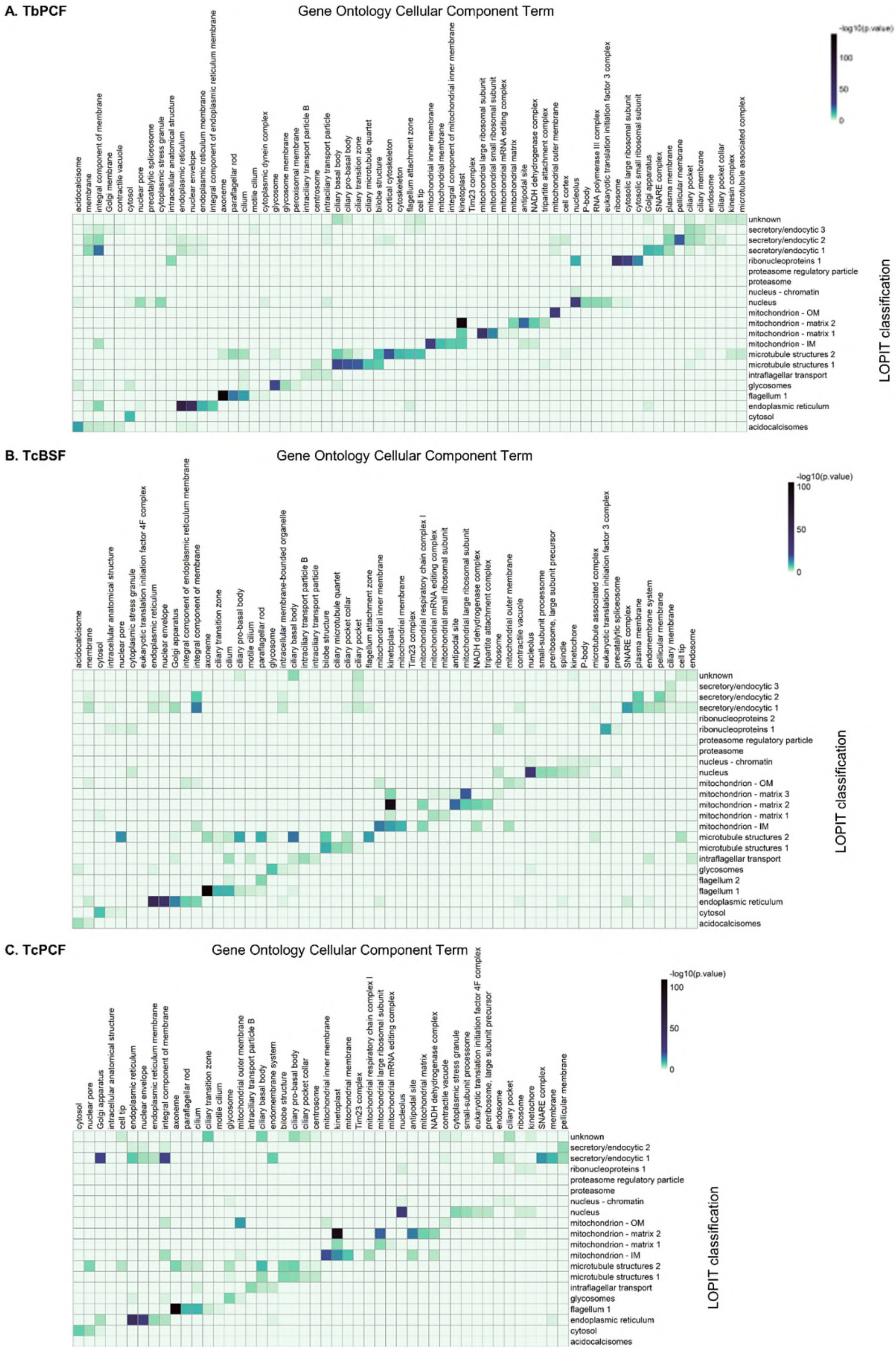

Supplementary Figure 8. Correlation of TAGM-MAP classifications with orthogonal approaches. Heatmap of GO CC term representation in TAGM-MAP classifications for (A) *T. brucei* PCF, (B) *T. congolense* BSF, and

(C) *T. congolense* PCF. hyperLOPIT compartments are on the Y-axis, GO CC terms are on the X-axis, colours are scaled by the -log10 (p-value) for the over-representation of GO CC terms in the indicated compartment versus the background proteome.

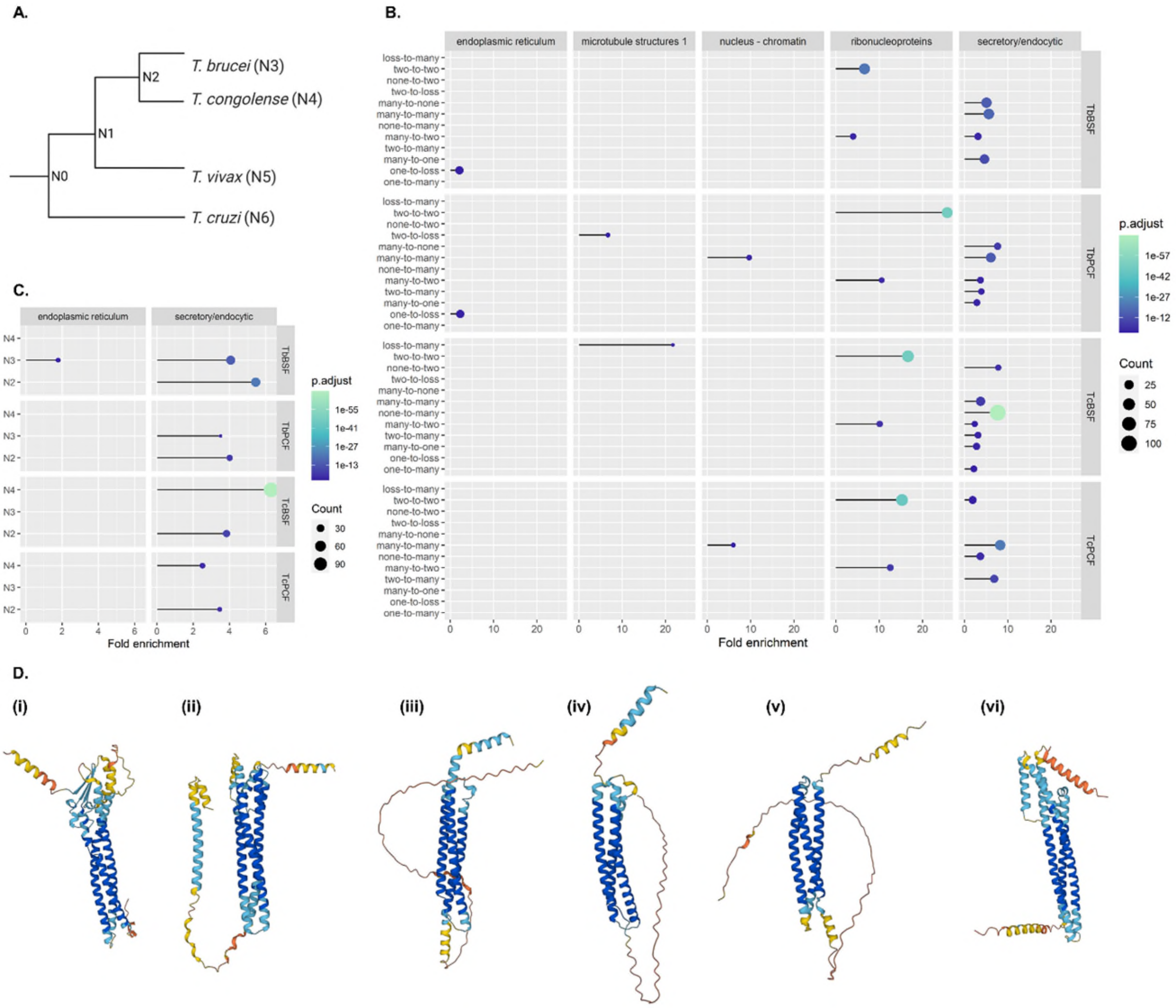

Supplementary Figure 9. Systematic inspection of evolutionary diversification in the spatial proteome. (A) Phylogenetic tree of trypanosome species used in OrthoFinder analysis – *T. brucei*, *T. congolense*, *T. vivax*, and *T. cruzi*. Node annotation indicates origin: N0: ancestral (n = 5923), N1: in all species except *T. cruzi* (n = 941), N2: *T. brucei* and *T. congolense* specific (n = 145), N3: *T. brucei* specific, N4: *T. congolense* specific (n = 564), N5: *T. vivax* specific (n = 1589), and N6: *T. cruzi* specific (n = 1086). Image created with BioRender.com. (B) Analysis of the categorical representation of gene counts (loss/ none/ one/ two/ many) in orthogroups between *T. brucei*-*T. congolense*. Lollipop chart showing the fold enrichment for over-represented *T. brucei*-*T. congolense* gene counts in subcellular compartments for indicated cell-type. Circle size is scaled by count of proteins in indicated compartment and colour by p-value for the over-representation of term versus the background proteome. (C) Lollipop chart showing the fold enrichment for over-represented origin annotation terms in subcellular compartments for indicated cell-type. Circle size is scaled by count of

proteins in indicated compartment and colour by p-value for the over-representation of term versus the background proteome. Enrichment analysis was performed using clusterProfiler (v4.0.5) ‘enricher’ function with default settings (hypergeometric test $p < 0.05$ with Benjamini and Hochberg adjustment). (D) AlphaFold predicted structure for i) Tb927.5.4630, ii) Tb927.7.4690, iii) Tb927.7.6490, iv) Tb927.7.6570 (ESP17), v) Tb927.7.6600 (SGM1.7), vi) Tb927.8.5910. Proteins are coloured according to per-residue confidence score (pLDDT). Dark blue: Very high (pLDDT > 90), Light Blue: Confident (90 > pLDDT > 70), Yellow: Low (70 > pLDDT > 50), Orange: Very low (pLDDT < 50). Images were taken from AlphaFold Protein Structure Database (https://alphafold.ebi.ac.uk/) under CC-BY-4.0 [26,27].

Supplementary Note 1. Concatenation of data from three experimental iterations: statistics and reproducibility

The computational approaches in LOPIT methodologies are based on assessment of protein co-fractionation patterns of lysed cell material dispersed across a physical gradient. Patterns of co-fractionation provides evidence of protein physical interaction and/or containment with objects that show a common distribution pattern across the gradients. The complexity of subcellular niches dictates that multiple parameters of cell lysis and gradient properties are used, and in the LOPIT method this is achieved by concatenating data from cell lysates dispersed across multiple different physical gradients. While the overall protein distribution patterns within these gradients might be similar, they do not function as replicates. Rather the reproducibility of protein co-fractionation provides evidence of the spatial co-location of the proteome. This strategy has been adopted by several different researchers utilising protein correlation profiles to determine co-location of proteins to date [28,29].

In this study, variation of gradient profiles is introduced in two ways. First, the lysis conditions used are non-identical between experiments, as described in Table S1. Second, the pooling of fractions is distinct between experiments. Pooling was guided by Western blot analysis (to maximise subcellular compartment resolution in individual runs) while deliberately varying resolution between runs where possible, with the constraint of protein availability in some cases. The pooling strategy for each experiment is described in Table S1. Whilst the different experimental iterations should not be considered replicates for the reasons described, the extent to which the 11-plex fractions cluster together across individual experiments can be visualised using PCA (Figure S2). This demonstrated consistency of the method between gradients although this is also not required.

Following the generation of the three 11-plex experiments per cell type, the data were concatenated to generate one 33-plex dataset per cell type. Concatenating the datasets is equivalent to assuming all datasets have equal weight and utilises all 33 fractions (features) for protein localisation prediction. As a result, this machine learning strategy gives full weight to all fractions from all datasets and therefore includes experiment-experiment variability in an unbiased framework.

Bayesian statistics were then used to analyse the concatenated data. The method employed models the full covariance between the different gradients and therefore accounts for the variation between them. Analysis of each gradient separately would not account for this co-variation. In order to assess the reproducibility of the classification outcomes between experiments, TAGM-MAP analysis was performed for each individual 11-plex dataset separately (Supplementary Data 8), in addition to the merged 33-plex datasets (Supplementary Data 9). Datasets were compared pairwise using the adjusted Rand index, which assigns a score of 0 if consistency is what is expected at random and 1 for perfect consistency. The Rand index was >0.85 for all pairwise comparisons, demonstrating good consistency between the classification outcomes of experimental iterations within each cell type (Figure S7). Note, to avoid inflating or deflating the adjusted Rand index due to "unknown" allocations, these were removed before comparison.

Supplementary References

1 Harsman, A. & Schneider, A. Mitochondrial protein import in trypanosomes: Expect the unexpected. *Traffic* **18**, 96-109, doi:10.1111/tra.12463 (2017).

2 Zíková, A., Verner, Z., Nenarokova, A., Michels, P. A. M. & Lukeš, J. A paradigm shift: The mitoproteomes of procyclic and bloodstream Trypanosoma brucei are comparably complex. *PLOS Pathogens* **13**, e1006679, doi:10.1371/journal.ppat.1006679 (2017).

3 Read, L. K., Lukeš, J. & Hashimi, H. Trypanosome RNA editing: the complexity of getting U in and taking U out. *Wiley Interdisciplinary Reviews: RNA* **7**, 33-51 (2016).

4 McDermott, S. M., Luo, J., Carnes, J., Ranish, J. A. & Stuart, K. The architecture of Trypanosoma brucei editosomes. *Proceedings of the National Academy of Sciences* **113**, E6476-E6485 (2016).

5 Aslett, M. *et al.* TriTrypDB: a functional genomic resource for the Trypanosomatidae. *Nucleic Acids Research* **38**, D457-D462, doi:10.1093/nar/gkp851 (2010).

6 Verner, Z. *et al.* Malleable mitochondrion of Trypanosoma brucei. *International review of cell and molecular biology* **315**, 73-151 (2015).

7 Makarov, A. A., Padilla-Mejia, N. E. & Field, M. C. Evolution and diversification of the nuclear pore complex. *Biochemical Society Transactions* **49**, 1601-1619 (2021).

8 Goos, C., Dejung, M., Janzen, C. J., Butter, F. & Kramer, S. The nuclear proteome of Trypanosoma brucei. *PLOS ONE* **12**, e0181884, doi:10.1371/journal.pone.0181884 (2017).

9 Clayton, C. & Estevez, A. The exosomes of trypanosomes and other protists. *Rna Exosome*, 39-49 (2010).

10 D'Archivio, S. & Wickstead, B. Trypanosome outer kinetochore proteins suggest conservation of chromosome segregation machinery across eukaryotes. *Journal of Cell Biology* **216**, 379-391 (2017).

11 Akiyoshi, B. & Gull, K. Discovery of unconventional kinetochores in kinetoplastids. *Cell* **156**, 1247-1258 (2014).

12 Nerusheva, O. O. & Akiyoshi, B. Divergent polo box domains underpin the unique kinetoplastid kinetochore. *Open biology* **6**, 150206 (2016).

13 Nerusheva, O. O., Ludzia, P. & Akiyoshi, B. Identification of four unconventional kinetoplastid kinetochore proteins KKT22–25 in Trypanosoma brucei. *Open biology* **9**, 190236 (2019).

14 Brusini, L., D'Archivio, S., McDonald, J. & Wickstead, B. Trypanosome KKIP1 dynamically links the inner kinetochore to a kinetoplastid outer kinetochore complex. *Frontiers in cellular and infection microbiology* **11** (2021).

15 Günzl, A. The pre-mRNA splicing machinery of trypanosomes: complex or simplified? *Eukaryotic cell* **9**, 1159-1170 (2010).

16 Gazestani, V. H. *et al.* A protein complex map of Trypanosoma brucei. *PLoS neglected tropical diseases* **10**, e0004533 (2016).

17 Erben, E., Chakraborty, C. & Clayton, C. The CAF1-NOT complex of trypanosomes. *Frontiers in genetics* **4**, 299 (2014).

18 Logan-Klumpler, F. J. *et al.* GeneDB—an annotation database for pathogens. *Nucleic acids research* **40**, D98-D108 (2012).

19 Maier, A. G. *et al.* The coatomer of Trypanosoma brucei. *Molecular and biochemical parasitology* **115**, 55-61 (2001).

20 Lee, S. H., Stephens, J. L. & Englund, P. T. A fatty-acid synthesis mechanism specialized for parasitism. *Nature Reviews Microbiology* **5**, 287-297 (2007).

21 Subota, I. *et al.* Proteomic analysis of intact flagella of procyclic Trypanosoma brucei cells identifies novel flagellar proteins with unique sub-localization and dynamics. *Molecular & cellular proteomics* **13**, 1769-1786 (2014).

22 Morga, B. & Bastin, P. Getting to the heart of intraflagellar transport using Trypanosoma and Chlamydomonas models: the strength is in their differences. *Cilia* **2**, 16, doi:10.1186/2046-2530-2-16 (2013).

23 Allmann, S. & Bringaud, F. Glycosomes: A comprehensive view of their metabolic roles in T. brucei. *The international journal of biochemistry & cell biology* **85**, 85-90 (2017).
24 Durrani, H., Hampton, M., Rumbley, J. N. & Zimmer, S. L. A global analysis of enzyme compartmentalization to glycosomes. *Pathogens* **9**, 281 (2020).
25 Hubert, L. & Arabie, P. Comparing partitions. *Journal of classification* **2**, 193-218 (1985).
26 Jumper, J. *et al.* Highly accurate protein structure prediction with AlphaFold. *Nature* **596**, 583-589 (2021).
27 Varadi, M. *et al.* AlphaFold Protein Structure Database: Massively expanding the structural coverage of protein-sequence space with high-accuracy models. *Nucleic acids research* **50**, D439-D444 (2022).
28 Krahmer, N. *et al.* Organellar Proteomics and Phospho-Proteomics Reveal Subcellular Reorganization in Diet-Induced Hepatic Steatosis. *Developmental cell* **47**, 205-221.e207, doi:10.1016/j.devcel.2018.09.017 (2018).
29 Lund-Johansen, F. *et al.* MetaMass, a tool for meta-analysis of subcellular proteomics data. *Nat Methods* **13**, 837-840, doi:10.1038/nmeth.3967 (2016).
