# Supplementary material for: Mapping diversity in African trypanosomes using high resolution spatial proteomics
Source: Nat Commun. 2023 Jul 21;14:4401. doi: 10.1038/s41467-023-40125-z (PMC10361982; doi:10.1038/s41467-023-40125-z)
Supplement: Supplementary file 2 — Description of Supplementary Files [file 41467_2023_40125_MOESM2_ESM.pdf]

Description of Additional Supplementary Files

File Name: Supplementary Data 1

Description: Additional experimental meta-data for hyperLOPIT experiments in *T. brucei* BSF, *T. brucei* PCF, *T. congolense* BSF, and *T. congolense* PCF.

File name: Supplementary Data 2

Description: Normalised protein quantitation data for three concatenated hyperLOPIT experiments in (A) *T. brucei* BSF, (B) *T. brucei* PCF, (C) *T. congolense* BSF, and (D) *T. congolense* PCF. Values are grouped according to biological iteration and TMT tagging.

File name: Supplementary Data

Description: Descriptions and identifiers of functionally related proteins. (A) Descriptions of sets of functionally related proteins of interest. Proteins were used to visually assess spatial resolution in *T. brucei* BSF and PCF hyperLOPIT datasets. (B) Gene identifiers and set names for functionally related proteins of interest. Proteins were used to visually assess spatial resolution in *T. brucei* BSF and PCF hyperLOPIT datasets.

File name: Supplementary Data 4

Description: Protein feature annotation (including biochemical properties, tSNE coordinates and unsupervised clustering) associated with spatial proteome data for (A) *T. brucei* BSF, (B) *T. brucei* PCF, (C) *T. congolense* BSF, and (D) *T. congolense* PCF.

File name: Supplementary Data 5

Description: Marker proteins for (A) *T. brucei* BSF and PCF TAGM-MAP and TAGM-MCMC classifications, (B) *T. congolense* BSF Novelty TAGM classification, (C) *T. congolense* PCF Novelty TAGM classification, (D) *T. congolense* BSF TAGM-MAP and TAGM-MCMC classifications, (C) *T. congolense* PCF TAGM-MAP and TAGM-MCMC classifications.

File name: Supplementary Data 6

Description: Novelty TAGM classifications of proteins to subcellular localisation in each 33-plex dataset (A) *T. congolense* BSF and (B) *T. congolense* PCF.

File name: Supplementary Data 7

Description: Extended summary of subcellular compartments represented in the spatial proteomes for each cell-type.

File name: Supplementary Data 8

Description: TAGM-MAP classifications of proteins to subcellular localisation for reproducibility analysis in each 11-plex dataset (individual experimental iterations) (A) *T. brucei* BSF experimental iteration (i) 1, (ii) 2, and (iii) 3, (B) *T. brucei* PCF experimental iteration (i) 1, (ii) 2, and (iii) 3, (C) *T. congolense* BSF experimental iteration (i) 1, (ii) 2, and (iii) 3, and (D) *T. congolense* PCF experimental iteration (i) 1, (ii) 2, and (iii) 3. Note that a more relaxed thresholding (localisation probability > 0.99) has been applied here compared with the classifications reported for the 33-plex datasets (Table S8).

File name: Supplementary Data 9
Description: TAGM-MAP classifications of proteins to subcellular localisation for spatial proteome definition in each 33-plex dataset (A) *T. brucei* BSF, (B) *T. brucei* PCF, (C) *T. congolense* BSF, and (D) *T. congolense* PCF.

File name: Supplementary Data 10
Description: TAGM-MCMC classifications of proteins to subcellular localisation in each 33-plex dataset (A) *T. brucei* BSF, (B) *T. brucei* PCF, (C) *T. congolense* BSF, and (D) *T. congolense* PCF. Note that no thresholding has been applied to the TAGM-MCMC allocations to yield classifications.

File name: Supplementary Data 11
Description: OrthoFinder analysis of *T. brucei*, *T. congolense*, *T. vivax*, and *T. cruzi* including a description of manual changes to hierarchical orthogroup classification.
